# Inboard advance of arc magmatism regulates mountain building in the Andes

**DOI:** 10.1038/s41467-026-71431-x

**Published:** 2026-04-11

**Authors:** Tomas N. Capaldi, Brian K. Horton, Chelsea Mackaman-Lofland, Facundo Fuentes, Gustavo Ortiz

**Affiliations:** 1 Scripps Institution of Oceanography, University of California San Diego, La Jolla, CA USA; 2 Jackson School of Geosciences, University of Texas at Austin, Austin, TX USA; 3 Department of Earth, Environmental, and Planetary Sciences, University of Tennessee, Knoxville, Knoxville, TN USA; 4 Independent Consultant, Buenos Aires, BA Argentina; 5 Departamento de Geofísica y Astronomía, Facultad de Ciencias Exactas, Físicas y Naturales, Universidad Nacional de San Juan, San Juan, SJ Argentina

**Keywords:** Tectonics, Stratigraphy, Structural geology

## Abstract

Debate continues over the mechanisms that govern the tempo and style of arc magmatism and retroarc deformation along ocean-continent plate boundaries. Here we integrate detrital and bedrock geo/thermochronological and geochemical records of Cenozoic arc magmatism with results on the spatiotemporal evolution of retroarc shortening and flexural subsidence to explore the interactions and feedbacks among subduction, magmatism, and crustal deformation in the southern Central Andes. A synthesis of igneous compositions, magmatic activity, thrust belt shortening, and sediment accumulation reveals recurring phases of increased arc magmatism, retroarc underthrusting, and foreland basin development over short ( < 10 Myr) timescales. Episodic advance of deformation was focused within discrete Andean tectonic provinces (of contrasting pre-Andean inheritance) that were sequentially activated toward the foreland along newly formed middle/upper-crustal décollements. Inboard migration of arc magmatism consistently preceded deformation advance, suggesting that fluid-assisted weakening above the subducting slab facilitated enhanced shortening and orogenic growth toward the cratonic interior.

## Abstract (web summary)

The study finds that when volcanic activity migrates inland, it weakens the Earth’s crust and kicks off quick episodes of mountain building, revealing a repeating pattern behind the rise of the Andes.

## Introduction

Interactions between arc magmatism and retroarc deformation shape topography, climate, and basin evolution in Andean-type orogenic systems along ocean-continent convergent margins. These relationships may represent responses to external drivers and/or internally coordinated processes that strongly influence crustal shortening, igneous activity, and basin subsidence. The mechanisms underlying time-space variations in tectonic and magmatic processes remain poorly understood. Most models linking the rates and magnitudes of arc magmatism and retroarc deformation have centered on cyclical behavior in orogens characterized by large-scale ( > 200–300 km) horizontal shortening, extreme crustal thickening ( > 60–70 km Moho depth), and episodic removal of thickened lower crust/lithosphere through foundering or delamination^1–4^. Such cycles occur over long timescales ( > 20–50 Myr) and preferentially affect only the largest and thickest orogenic systems. However, potential internal cycles of shorter duration ( < 10 Myr) may also operate during mountain building, particularly in regions of lower-magnitude ( < 50–150 km) shortening. Here we hypothesize that short-term orogenic phases linked to shifts in thermal and rheological conditions help regulate dynamic interactions among subduction, arc magmatism, and retroarc deformation.

Evaluating Andean-type orogenesis requires consideration of the diverse mechanisms that shape spatial-temporal patterns of arc magmatism and retroarc shortening. Arc migration toward the continental interior has been associated with heightened magmatic activity along subduction margins^5^. Such landward migration expands slab dewatering within the mantle wedge, enhancing fluid flux and hydration of the overriding foreland lithosphere^6^, which may further induce crustal weakening and strain localization^7^.

A range of internal and external mechanisms within the upper and lower plates may account for inboard advance of arc magmatism^8^. Internally, within the overriding plate, arc advance may be a product of horizontal shortening^9^ and/or forearc subduction erosion^10,11^. In addition, arc advance may be driven by external forces that affect the subducting plate, including accelerated plate convergence^12^ or low-angle (flat slab) subduction^13–16^. Although slab shallowing is commonly attributed to subduction of buoyant (i.e., younger or thicker) oceanic lithosphere, several studies indicate a poor correlation among oceanic slab age, crustal thickness, seafloor asperities, and slab dip^17,18^. Alternatively, slab shallowing may be driven by the position of thick continental lithospheric roots that promote hydrodynamic suction (or negative pressure) associated with corner flow in the mantle wedge between the subducting and overriding plates^19,20^. Because the aforementioned interactions vary across spatial and temporal scales, it is important to identify the processes responsible for arc migration and assess their impact on short-term (< 10 Myr) changes in magmatism and retroarc orogenesis within Cordilleran systems.

**Fig. 1:**
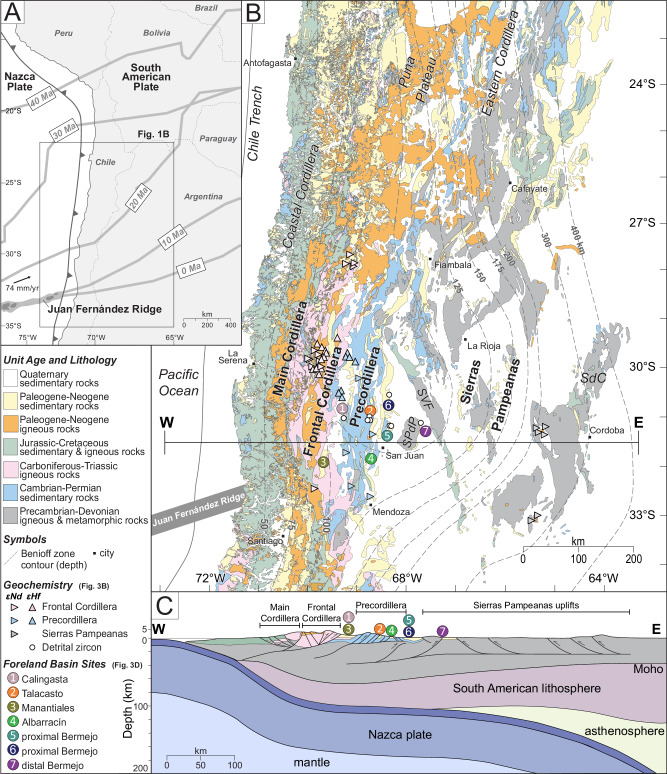
Tectonic framework of the southern Central Andes. **A** Inset map of western South America and the projected position of the subducted Juan Fernandez Ridge from 40 to 0 Ma^88^. **B** Geologic map of the southern Central Andes showing major tectonic provinces, basin study sites, geochemistry sample locations, and depth to the oceanic Nazca slab (dashed Benioff contour lines)^89^. **C** East-West profile of Nazca-South America plate boundary illustrating the crustal/lithospheric architecture of the Pampean flat-slab subduction segment and the Andean orogen, including the Main Cordillera, Frontal Cordillera, Precordillera, and Sierras Pampeanas (after^55,75^). SdC Sierra de Cordoba, SPdP Sierra de Pie de Palo, SVF Sierra de Valle Fertíl.

In this study, we compare the spatial, temporal, and compositional variations of arc magmatism in the southern Central Andes with geo/thermochronological, geochemical, and stratigraphic datasets to better understand the geodynamic links among subduction, magmatism, and Andean orogenesis. The modern Pampean segment (27–33°S) of the Nazca-South American plate boundary (Fig. 1) is characterized by flat slab subduction, a ~ 500 km long gap in active volcanism, and foreland basement uplifts of the Sierras Pampeanas located >700 km inboard of the trench (Fig. 1A)^15,21–24^. Our analysis focuses on the past 30 Myr of mountain building in western Argentina, a region characterized by persistently arid climatic conditions^25,26^, which enables the isolation of tectonic signals associated with orogenic wedge behavior. Synthesizing records of Andean arc magmatism and retroarc deformation provides an opportunity to explore feedbacks between subducting and overriding plates and identify potentially cyclical mechanisms that regulate mountain building along convergent plate boundaries.

## Results

### Magmatic arc activity and composition

**Fig. 2:**
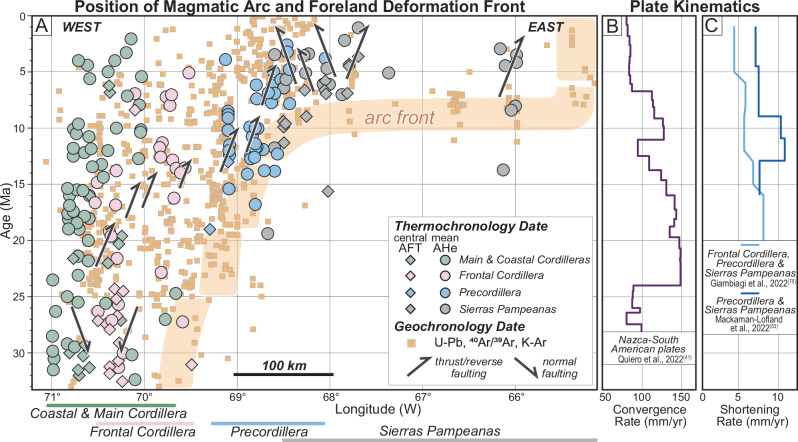
Cenozoic position of arc magmatism and foreland deformation during Nazca-South America plate convergence. **A** Time-distance plot of geo/thermochronological dates at 27–33°S illustrating progressive eastward advance of arc magmatism and retroarc foreland deformation. Black arrows show time-space constraints for deformation in the arc and retroarc regions. **B** Trench-normal convergence rates at 30°S. **C** Shortening rates in the Andean retroarc foreland.

**Fig. 3:**
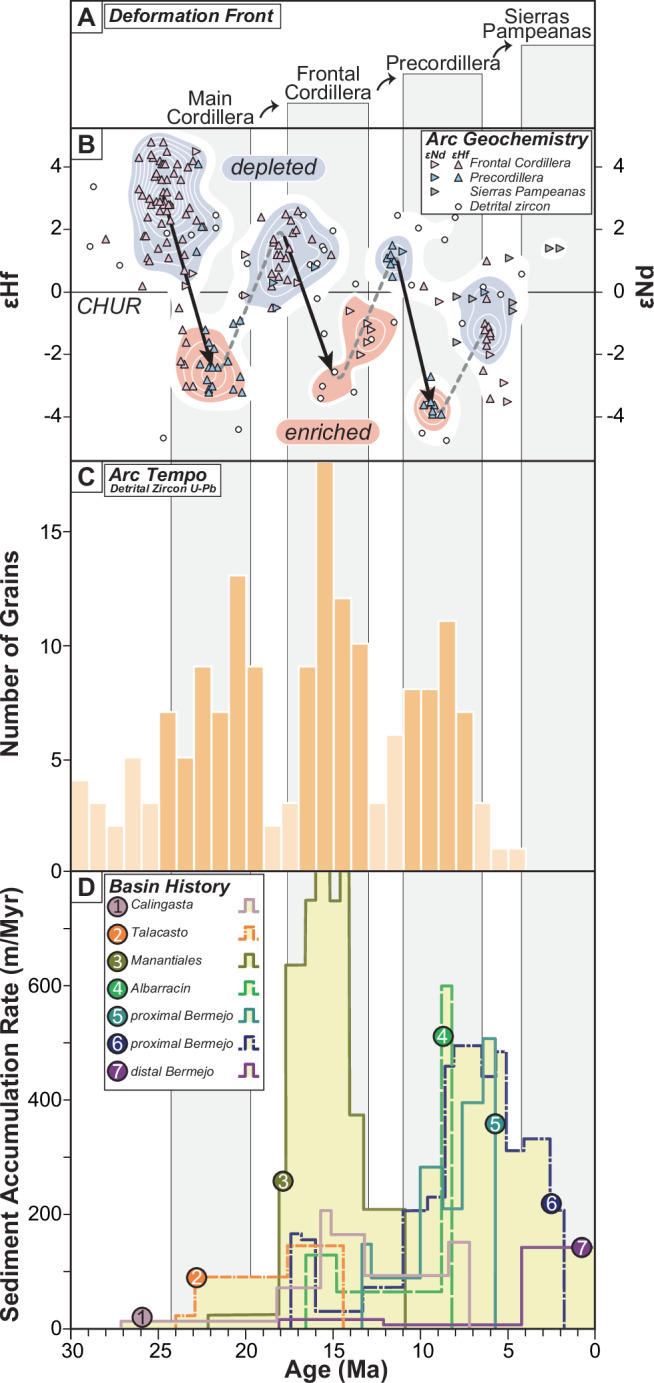
Temporal variations in Andean arc magmatism and retroarc foreland processes. **A** Four main episodes ( < 10 Myr duration) of Andean shortening (vertical gray bars) illustrating sequential eastward advance of retroarc deformation across the major tectonic provinces (Fig. 2). **B** Episodic changes in magmatic arc composition recorded by zircon εHf and whole rock εNd isotopes (shading and contours show depleted (blue) vs. enriched (red) sources). Data from^24,29,85,86^. **C** Temporal trends in magmatic activity tracked by variations in detrital zircon U-Pb ages. Data from^24^. **D** Sediment accumulation rates in the retroarc foreland basin for different sites: 1: Calingasta^57^; 2: Talacasto^46^; 3: Manantiales^43,90,91^; 4: Albarracín^46,92^; 5: proximal Bermejo^52^; 6: proximal Bermejo^93^; 7: distal Bermejo^57^ (Fig. 1).

To assess time-space patterns of arc magmatism and retroarc crustal shortening, we compile crystallization ages of Cenozoic igneous rocks along an arc-normal east-west profile (Fig. 2A). These regional patterns in arc magmatism (Fig. 2A) are compared with arc tempo and magmatic compositional data registered in whole-rock and zircon minerals (Fig. 3B, C). Geochronological results paired with εHf and εNd isotopic compositions provide valuable temporal recorders of continental arc evolution that are sensitive to upper-crustal composition and crystallization conditions. Positive εHf and εNd values reflect juvenile crustal material originating from melts of depleted (juvenile) mantle origin, whereas negative isotopic values signify enriched melts derived from older, recycled (evolved) crustal material^27–29^.

The geochemical match between detrital zircon εHf values and those of spatially resolved igneous bedrock (Fig. 3A) strengthens confidence in their provenance and demonstrates their value as a complementary tool for tracking magmatic evolution through time. Detrital zircon age distributions for continental magmatic arcs and their associated basins are robust temporal indicators of arc activity, suitable for tracking rates of zircon-bearing arc-magma production and identifying punctuated high-volume magmatic events in the form of flare-ups or high-flux events (HFEs)^30–32^.

Oligocene arc magmatism (33–25 Ma) was focused over a narrow (~100 km wide) zone within the Main (Principal Cordillera (Fig. 2A). Compositionally, these magmas exhibit positive εHf and εNd values, indicative of depleted mantle wedge sources (Fig. 3B). By 25 Ma, the arc advanced eastward, forming a broader (~200 km wide) zone that spanned the Main and Frontal Cordilleras (Fig. 2A). During the late Oligocene to early Miocene (25–19 Ma) zircon εHf trends delineate an isotopic pull-down toward enriched magma sources contemporaneous with increased arc activity recorded by greater proportions of detrital zircon ages (Fig. 3B, C). From 19 to 13 Ma, early to middle Miocene magmatism expanded eastward into the Precordillera (Fig. 2A). At this time, zircon εHf and whole-rock εNd results exhibited further isotopic pull-down from positive (depleted) to negative (enriched) values coeval with increased arc activity (Fig. 3B, C). A broad swath of magmatism was activated at 13–6 Ma, indicating rapid eastward advance ( > 400 km) of magmatism toward the craton. Arc geochemical data again recorded a shift to lower (enriched) values synchronous with heightened arc magmatism (Fig. 3B, C). At ~5 Ma, a notable cessation of arc magmatism occurred within the Main and Frontal Cordilleras (71–69°W), the former locus of Neogene magmatism.

Coupled geochemical and age data from igneous and detrital samples exhibit a progressive decrease in zircon εHf and whole-rock εNd values over the past 30 Myr (Fig. 3B), reflecting the cumulative effects of crustal thickening driven by sustained shortening during Andean orogenesis. When considered alongside bedrock sample locations (Fig. 2A), a spatial relationship emerges: relatively asthenospheric (juvenile) compositions are preferentially situated near the trench, while more lithospheric (evolved) contributions are concentrated toward the craton (Fig. 3B). This spatial gradient manifests as temporal variability, as the magmatic arc migrated eastward into older continental lithosphere with distinct isotopic signatures. Superimposed on this long-term (30 Myr) trend are higher-frequency phases (5–8 Myr) marked by transient shifts in εHf and εNd values. These phases represent background arc magmatism sourced from juvenile mantle, periodically perturbed during isotopic pull-downs that recorded increased crustal contamination and partial melting during phases of shortening and underthrusting (Fig. 3B). At the onset of each pull-down, a 1–2 Myr temporal overlap of depleted and enriched values suggests magma mixing processes during transitions in source contributions^33^. Together, these patterns underscore the dual influence of arc migration into older lithosphere and progressive crustal thickening in shaping the isotopic evolution of Andean magmatism.

### Retroarc shortening and flexural response

Structural kinematic records, low-temperature thermochronologic data, and foreland subsidence histories collectively document the timing and style of retroarc shortening, exhumation, and crustal loading. First, thermochronometric results constrain bedrock cooling in response to upper crustal erosion (Fig. 2A). Apatite fission track (AFT) and (U-Th)/He (AHe) thermochronometric dates are based on temperature-dependent diffusive loss of radiogenic daughter products or fission damage trails and record mineral cooling below ~40–120 °C. These systems are sensitive to the exhumation history of rocks within approximately 6 km of the surface^34^. Second, eastward migration of the retroarc foreland basin during construction of the southern Central Andes provides an independent means of tracking deformation advance^35,36^. Third, sediment accumulation histories along an arc-normal transect of foreland basin sections are leveraged to highlight spatial-temporal changes in the flexural response to retroarc shortening during sequential activation of discrete contractional systems toward the foreland (Fig. 3D).

The upper crustal tectonic regime varied throughout Cenozoic subduction. During the middle Eocene to early Oligocene, intra-arc transtensional faulting governed basin formation^37–39^. Subsequent late Oligocene–early Miocene shortening accompanied increased convergence along the subduction margin during breakup of the oceanic Farallon plate into the Nazca and Cocos plates (Fig. 2B)^40,41^. This tectonic shift coincided with the main phase of Andean shortening, which was accommodated in the Main (Principal) Cordillera by thin-skinned shortening and inversion of Triassic to Paleogene extensional and retroarc basin fill^39,42^.

Rapid foreland subsidence in western basin sites (i.e., Calingasta, Talacasto, and Manantiales) initiated by 24–22 Ma in response to shortening in the Main Cordillera^26,43^. Thereafter, deformation advanced eastward to deeper structural levels of the Frontal Cordillera, where reverse faults exhumed mechanical basement composed of a > 3 km thick panel of Permian-Triassic rhyolitic and granitic rocks (Choiyoi Group) associated with the pre-Andean Cuyo rift system^44,45^. Accelerated sediment accumulation at 19–13 Ma recorded foredeep development during shortening, uplift, and thrust loading in the Frontal Cordillera^43,46^.

Thermochronology and sediment provenance data reveal an eastward progression of exhumation within the orogenic wedge from the Andean hinterland (Main Cordillera and Frontal Cordillera) to the thin-skinned Precordillera thrust belt by 16 Ma^47,48^. Precordillera shortening was characterized by imbricate faults that deformed a Paleozoic stratigraphic wedge above a regional décollement along the basement-cover interface^49,50^. From 13 to 7 Ma, the foredeep migrated eastward while 65–100 km of E-W shortening was accommodated in the Precordillera^36,47,49,51,52^. Coeval cooling in the hinterland suggests passive exhumation as the trailing thrust sheet was translated over a mid-crustal ramp that links eastward into the Precordillera décollement^53,54^.

By 6 Ma, shortening-induced exhumation advanced eastward into the Sierras Pampeanas, where intraforeland basement-cored uplifts developed above crustal-scale reverse faults^15,55,56^. Diminished sediment accumulation after ~7 Ma in the eastern foreland reflects a transition to broken foreland conditions, as Sierras Pampeanas basement uplifts partitioned the once-continuous foreland basin into disconnected depocenters^57,58^.

## Discussion

### Multi-phase model

**Fig. 4:**
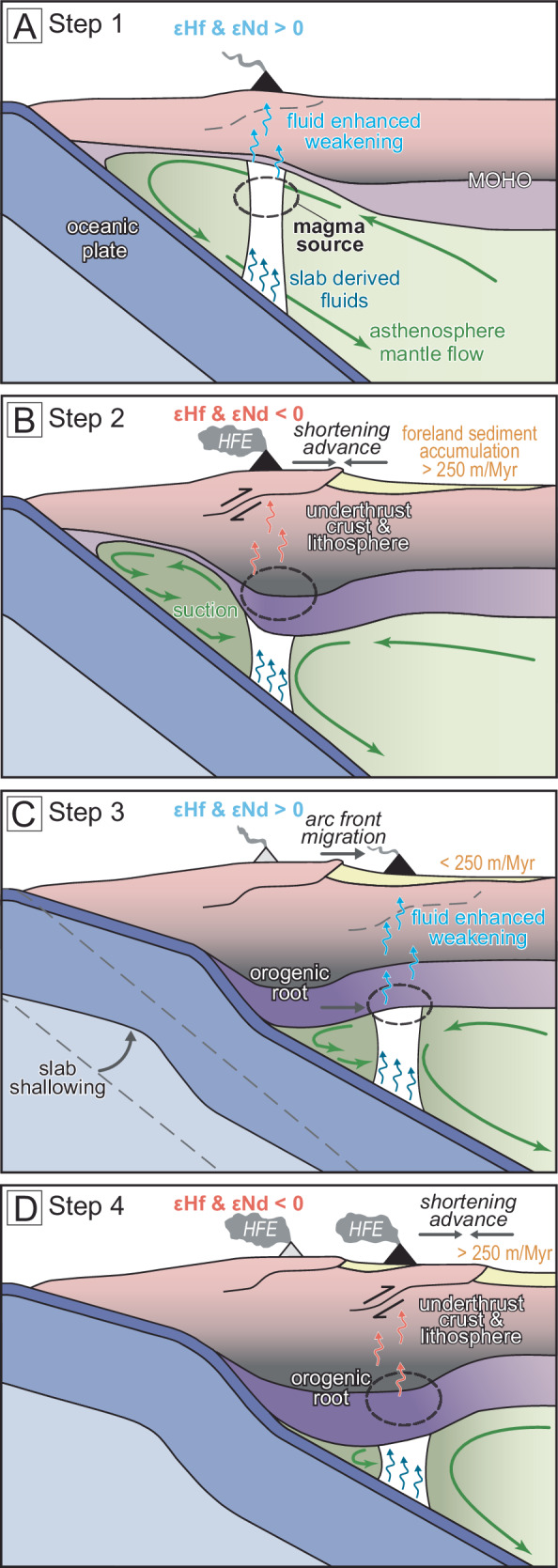
Schematic cross sections illustrating successive stepwise variations in arc magmatism, magmatic sources, retroarc deformation, and interactions between subducting and overriding plates. **A** Subduction (neutral to retreating mode) causes dehydration melting with ascending melts and fluids affecting the overriding plate. **B** Fluid-induced weakening promotes retroarc shortening with underthrusting and crustal thickening that fuels high-flux event (HFE) arc magmatism and increased corner flow in the mantle wedge. **C** Reduced pressure (suction) in the mantle wedge leads to slab shallowing, which drives inboard arc migration with ascending melts and fluids affecting inboard continental regions. **D** Weakening of continental interior triggers a new phase of deformation advance involving renewed shortening, underthrusting, and crustal thickening associated with enhanced arc magmatism and flexural subsidence in the retroarc foreland basin.

With the southern Central Andes as a template, we propose a process-based, multi-step model for contractional orogens (Fig. 4) that accounts for the distribution and tempo of subduction-related magmatism in association with discrete short-lived ( < 10 Myr) phases of shortening.

Step 1. The conceptual model commences with subduction-induced dehydration of serpentinized oceanic lithosphere, which modifies the strength of the overriding continental plate (Fig. 4A). Continuous incorporation of water and other volatiles lowers mantle wedge viscosity^59^, fostering rapid small-scale convection that routes high-temperature asthenosphere toward retroarc regions^60,61^. In turn, the elevated temperatures enable rapid thermal weakening, raising the brittle-ductile transition and decreasing the differential stress required for failure^62,63^. Intensified input of slab-derived fluids and associated melts further weakens the overlying crust, a process proposed to enhance crustal deformation^7,64–68^. Fluid infiltration into the crust could be facilitated by preexisting structures and suture boundaries^69^.

Step 2. Earlier fluid-induced weakening promotes the activation of retroarc shortening with attendant underthrusting and crustal thickening that fuels high-flux magmatism (Fig. 4B). The high-flux events (HFEs) are marked by isotopically enriched magmatic sources (isotopic pull-downs) relative to baseline magmatism characterized by depleted mantle wedge signatures. The enriched isotopic signatures can be attributed to the interaction of mantle-derived magmas with thickened continental crust, involving greater contamination and/or partial melting within the lower crust (MASH zone)^10,28,29^. Underthrusting of melt-fertile continental lithosphere beneath the magmatic arc is a common trigger for HFEs^5,9,70–72^. Retroarc shortening in the fold-thrust belt also induces topographic loading and flexural subsidence, such that accelerated sediment accumulation in the foreland basin tracks progressive crustal thickening and steadily migrates in tandem with deformation front advance toward the craton^57^.

Step 3. Continued retroarc shortening and possible magmatic underplating produce a thickened continental lithosphere (Fig. 4B). The growing orogenic root impedes mantle wedge circulation, reducing pressure and creating suction between the downgoing slab and overriding plate^19^. This pressure reduction leads to slab shallowing^20,73^ that narrows the mantle wedge as asthenospheric material is evacuated and the magmatic arc advances inboard (Fig. 4C)^64^. Slab-derived fluids further reduce the viscosity of the asthenospheric mantle and therefore viscous resistance to slab subduction, enabling inboard advance of both the shallowing slab segment and mantle wedge^74^.

Step 4. The lateral migration of slab dewatering leads to continuous inboard hydration and weakening of continental lithosphere, incrementally triggering deformation advance (Fig. 4D). Further shortening is transferred to a series of separate, successively activated décollements in the middle and upper crust, which develop in sequence toward the foreland, with diachronous deactivation of trailing hinterland thrust systems^75^. The locus of flexural subsidence in the retroarc foreland basin migrates inboard along with the advancing deformation front^58^. Continued crustal underthrusting initiates a new phase of high-flux magmatism, producing an isotopically enriched (negative εHf and εNd) magmatic signature (Fig. 4D). Sustained growth of a thick orogenic root leads to further mantle wedge suction and slab shallowing, initiating a new cycle (Steps 1–4) of arc broadening, inboard arc advance, and retroarc shortening along newly developed décollements.

### Implications for Andean-type (Cordilleran) orogenic systems

Comparisons of high- vs. low-shortening orogens suggest that magmatic arc advance and fluid-assisted retroarc weakening are vital components of Cordilleran mountain building. The Cretaceous-Paleogene Sevier and Laramide belts of western North America^4,76,77^ and Late Cretaceous to modern Andes^1,71,78^ exhibit recognizable time-space connections between arc magmatism and retroarc deformation. In both cases, the magmatic arcs expanded systematically to widths of ~75–200 km while advancing >300–1500 km toward the foreland (without considering palinspastic restoration of crustal shortening). The foreland deformation front advanced in step with an expanded magmatic system (Fig. 2), contemporaneous with progressive slab flattening. Where isotopic data exist, phases of protracted shortening coincide with HFEs in the magmatic arc, which show geochemical signatures indicative of enhanced crustal melt contributions. The frequency of HFEs serves as a proxy for arc tempo, with magmatic duration and periodicity varying across orogenic systems.

In high-shortening systems ( > 200–300 km) with extreme crustal thickening ( > 60–70 km)—such as the Sevier-Laramide orogen and Central Andes (15–25°S)—HFEs occur at intervals of ~25–45 Myr, coeval with punctuated shortening episodes^1,3,4,16,71,79^. In contrast, low-shortening systems ( < 50–150 km), including the southern Andes (25–50°S) and Mexican fold-thrust belt, exhibit magmatic arc tempos (Fig. 3) that reflect short-lived ( < 10 Myr) orogenic phases^22,77^. Such variations in the periodicity of magmatism and shortening may reflect preexisting structural and stratigraphic heterogeneities within the overriding continental crust. In particular, the irregular thickness and distribution of Phanerozoic retroarc strata may either promote or inhibit the advance of fold-thrust systems and décollements, depending on the presence of mechanical anisotropies within the cover strata or along the basement-cover interface^68^. The high-shortening segments of the North and South American Cordilleras are underlain by >10 km thick stratigraphic wedges— nearly double the thickness observed in low-shortening segments^77,80^. In this context, limited stratigraphic cover corresponds with punctuated ( < 10 Myr) deformation and high-flux magmatism, whereas thicker stratigraphic cover correlates with protracted ( > 20 Myr) thrust-belt shortening and longer-duration HFEs. The longevity of orogenic phases is further modulated by the inherited structural and stratigraphic framework of the continental lithosphere. Comparable patterns of arc migration and retroarc shortening point to periodic dehydration of the subducting slab, which regulates the strength of the overriding lithosphere and may thus focus sequential deformation activation toward the craton (Fig. 4).

Flat slab subduction is equally recognized as a key tectonic process in both high- and low-shortening systems. Slab shallowing has been attributed to subduction of thick oceanic lithosphere or bathymetric asperities in which greater buoyancy diminishes slab dip (e.g.^81,82^). Others suggest that the size of these features is insufficient to initiate slab flattening (e.g.^17,18^). However, oceanic ridges and seamounts are typically associated with strongly hydrated lithosphere with concentrated deformation and fluid expulsion^83,84^. We suggest that slab dewatering may induce fluid weakening and strain localization in the overriding plate^69^. In turn, focused crustal thickening and generation of a thick orogenic root within the overriding plate promotes hydrodynamic suction (or negative pressure) in the mantle wedge, inducing systematic slab flattening^19,20^. This proposed dynamic link involving dewatering of the subducted oceanic plate, fluid-enhanced weakening, and thickening of the continental plate (Fig. 4) offers an additional potential mechanism for slab shallowing. Ultimately, fluid-mediated weakening, generation of lithospheric roots, and slab flattening emerge as central mechanisms driving the spatial and temporal patterns of deformation and magmatism in Cordilleran orogenic systems.

## Methods

The spatial distribution of magmatism is constrained by an extensive compilation (n = 741; Supplemental Data 1) of Eocene to Pleistocene bedrock radiometric ages at 27°–32.5°S (Fig. 2A)^82^. Data curation involved calculating a mean age for igneous samples and omission of duplicate and detrital ages. The spatial and temporal distributions of igneous ages are plotted on Supplemental Fig. 1. Whole-rock εNd data were derived from an igneous bedrock compilation (n = 28)^85^, whereas the zircon εHf values are from both igneous bedrock (n = 128)^86^ and detrital zircon samples (n = 44)^24^ spanning the Chile-Argentina hinterland and retroarc regions to the east (Fig. 3B; Supplementary Data 2 and Supplementary Data 3). All geochemical data (Fig. 3B) were plotted using the Hafnium Plotter software^87^. The detrital zircon U-Pb age distribution representing Andean arc activity comes from a Phanerozoic compilation (Fig. 3C)^24^. Compiled mean AHe (n = 103) and central AFT (n = 53) thermochronology dates are an aggregate of both crystalline and sedimentary samples across the southern Central Andes at 27–32.5°S (Fig. 2A; Supplementary Data 4, 5). Filtering of apatite (U-Th-Sm)/He and fission track dates adheres to rejection criteria that excludes anonymously low He concentrations, non-reset dates, high date dispersion, date outlier, and overlap with igneous age or proximal hydrothermal activity. The spatial distributions of thermochronometric ages are plotted in Supplemental Fig. 2, while single-grain ages of the mean AHe samples (Fig. 2A) are plotted in Supplemental Fig. 3. Structural constraints on faulting in the arc and retroarc regions are supported by synorogenic stratigraphic, structural, cross-cutting relationships, thermochronology data, and thermokinematic modeling (Fig. 2A; Supplementary Data 6). Sediment accumulation rates are calculated from seven basin locations (Fig. 1) with previously measured stratigraphic sections and associated age constraints (Fig. 3D; Supplementary Data 7).

## Supplementary information

Supplementary Information

Description of Additional Supplementary Files

Supplementary Data 1-7

Transparent Peer Review file

## Data Availability

The geochronology, thermochronology, geochemical, stratigraphic, and structural data generated in this study are provided in the supplementary information files.
