# Supplementary material for: Inboard advance of arc magmatism regulates mountain building in the Andes
Source: Nat Commun. 2026 Apr 11;17:5082. doi: 10.1038/s41467-026-71431-x (PMC13247209; doi:10.1038/s41467-026-71431-x)
Supplement: Supplementary file 1 — Supplementary Information [file 41467_2026_71431_MOESM1_ESM.pdf]

Inboard advance of arc magmatism regulates mountain building in the Andes

Tomas N. Capaldi[1*], Brian K. Horton[2], Chelsea Mackaman-Lofland[3], Facundo Fuentes[4], and Gustavo Ortiz[5]

[1] Scripps Institution of Oceanography, University of California San Diego, La Jolla, CA 92093, USA
[2] Jackson School of Geosciences, University of Texas at Austin, Austin, TX 78712, USA
[3] Department of Earth, Environmental, and Planetary Sciences, University of Tennessee, Knoxville, TN 37996, USA
[4] Independent Consultant, Buenos Aires, Argentina
[5] Departamento de Geofísica y Astronomía, Facultad de Ciencias Exactas, Físicas y Naturales, Universidad Nacional de San Juan, Meglioli 1160 S, San Juan, Argentina
[*]Corresponding author email: tcapaldi@ucsd.edu

Supplementary Data Table of Contents:
Supplementary Data 1: Igneous Geochronology Date Compilation (Fig. 2A; Supplemental Figure 1)

Supplementary Data 2: Compilation of Bedrock Nd isotope data (Fig. 1B; Fig. 3B)

Supplementary Data 3: Compilation of Bedrock and Detrital Zircon Lu-Hf data (Fig. 1B; Fig. 3B)

Supplementary Data 4: Apatite (U-Th-Sm)/He Thermochronology Date Compilation (Fig. 2A; Supplemental Figure 2; Supplemental Figure 3)

Supplementary Data 5: Apatite Fission Track Thermochronology Date Compilation (Fig. 2A; Supplemental Figure 2)

Supplementary Data 6: Deformation Front constraints from synorogenic stratigraphic data, structural and cross-cutting relationships, thermochronology data, and thermokinematic modeling (Fig. 2A).

Supplementary Data 7: Chronostratigraphic data used for Retroarc foreland basin sediment accumulation histories (Fig. 3D). 1: Calingasta; 2: Talacasto; 3: Manantiales; 4: Albarracín; 5: proximal Bermejo (Sierra Villicum); 6: proximal Bermejo (Mogna Anticline); 7: distal Bermejo (Ampacama)

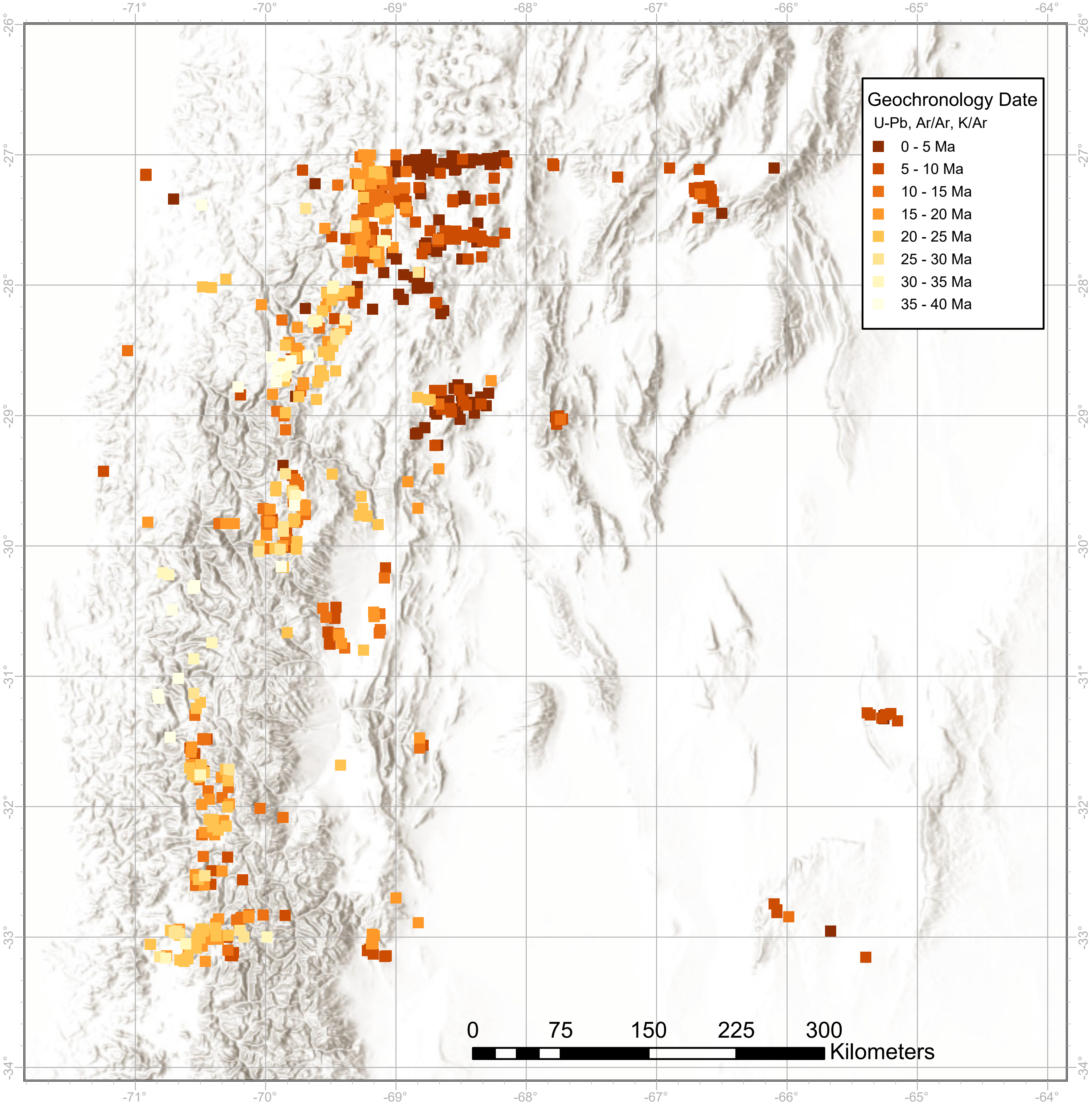

Supplemental Figure 1: Spatial and temporal variation in Andean arc magmatism. Data compilation of U-Pb, Ar/Ar and K/Ar ages from Pilger, 2024.

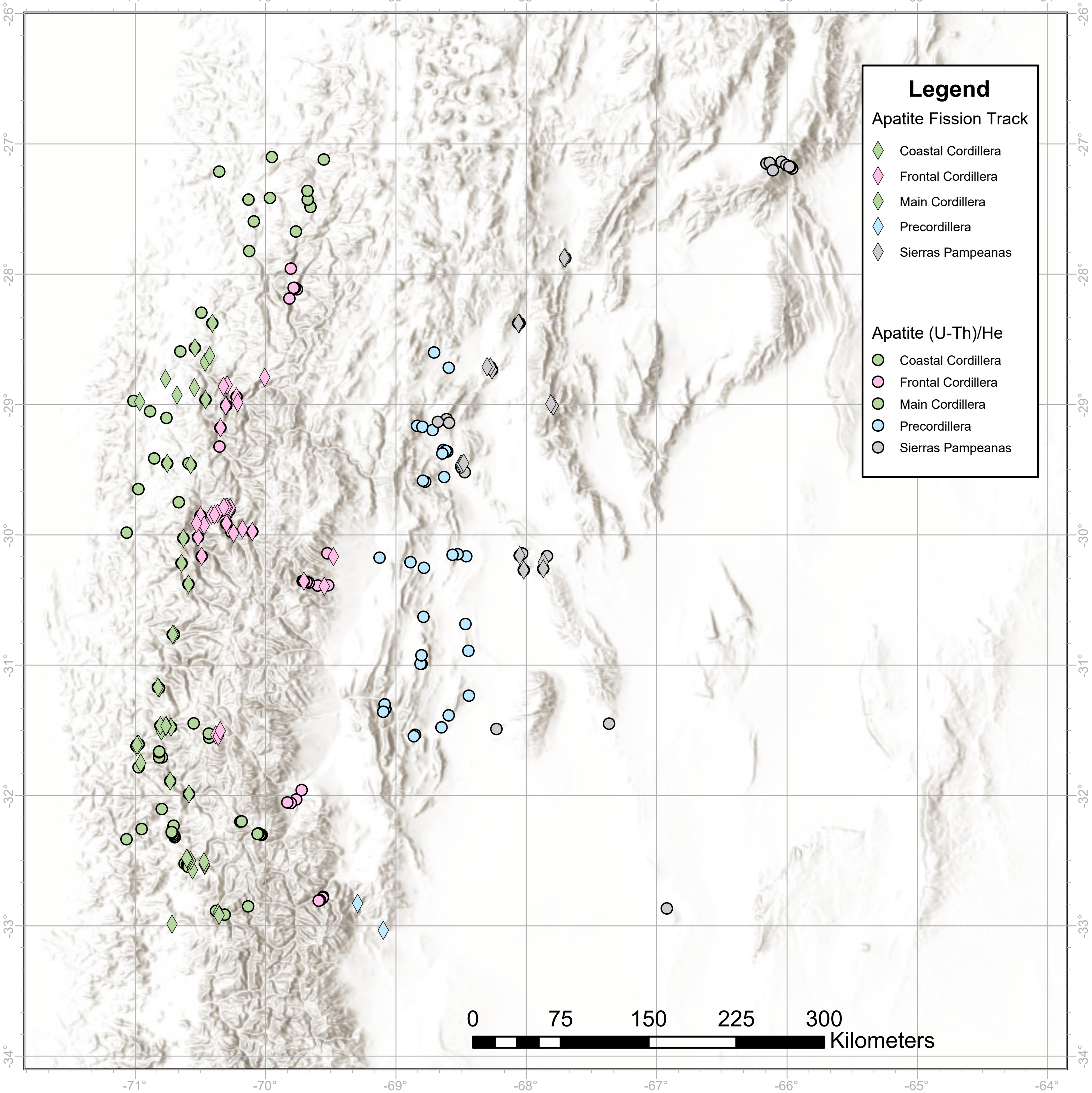

Supplemental Figure 2: Spatial distribution in Andean thermochronological dates. Data compilation of apatite (U-Th)/He dates from: Lobens et al., 2013; Bense et al., 2013; Levina et al., 2014; Hoke et al., 2015; Fosdick et al., 2015; Ortiz et al., 2015, 2021; Lossada et al., 2017; Piquer et al., 2017; Rodriquez et al., 2018; Stevens-Goddard and Carrapa, 2018; Maydagan et al., 2020; Mackaman-Lofland et al., 2020, 2022, 2024; Stalder et al., 2020; Plonka et al., 2023. Data compilation of apatite fission track dates from: Coughlin et al., 1998; Avila et al., 2005; Farias et al., 2008; Sato et al., 2015; Fosdick et al., 2015; Murillo et al., 2017; Rodriguez et al., 2018; Stevens-Goddard and Carrapa, 2018; Maydagan et al., 2020; Stalder et al., 2020; Ortiz et al., 2021.

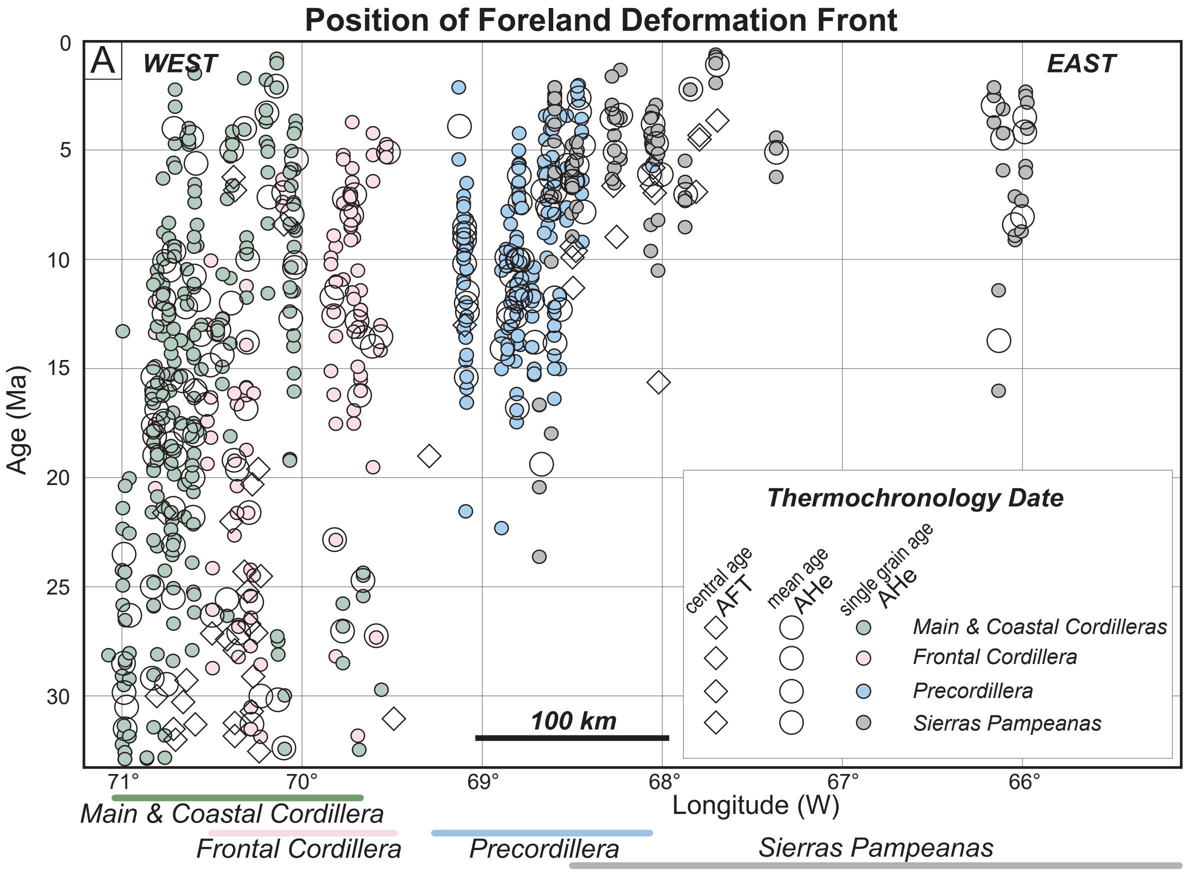

Supplemental Figure 3: Time-distance plot of single grain and mean/central age thermochronological dates at 27–33°S illustrating progressive eastward advance of retroarc foreland deformation. Data compilation of apatite (U-Th)/He dates from: Lobens et al., 2013; Bense et al., 2013; Levina et al., 2014; Hoke et al., 2015; Fosdick et al., 2015; Ortiz et al., 2015, 2021; Lossada et al., 2017; Piquer et al., 2017; Rodriquez et al., 2018; Stevens-Goddard and Carrapa, 2018; Maydagan et al., 2020; Mackaman-Lofland et al., 2020, 2022, 2024; Stalder et al., 2020; Plonka et al., 2023. Data compilation of apatite fission track dates from: Coughlin et al., 1998; Avila et al., 2005; Farias et al., 2008; Sato et al., 2015; Fosdick et al., 2015; Murillo et al., 2017; Rodriguez et al., 2018; Stevens-Goddard and Carrapa, 2018; Maydagan et al., 2020; Stalder et al., 2020; Ortiz et al., 2021.

References Cited in Supplementary Data

Almonacid, T., 2007, Geología de la Zona de Alteración Hidrotermal de Domeyko y del Yacimiento de Cobre Dos Amigos, Región de Atacama, Chile., Tesis para optar al grado de Magíster en Ciencias Mención Geología (Inédito), Universidad de Chile, Departamento de Geología: 114 p.,

Alonso, M.S.; Limarino, C.O.; Litvak, V.D.; Poma, S.M.; Suriano, J.; Remesal, M.B. 2011. Paleogeographic, magmatic and paleo environmental scenarios at 30ºS during the Andean orogeny: Cross sections from the volcanic-arc to the orogenic front (San Juan, Argentina). In Cenozoic Geology of the Central Andes of Argentine (Salfity, J.A.; Marquillas, R.A.; editors). SCS Publisher: 23-45. Salta

Álvarez, J., Mpodozis, C., Blanco-Quintero, I., García-Gasco, A., Arriagada, C., Morata, D., 2013, U/Pb ages and metamorphic evolution of the La Pampa Gneisses: Implications for the evolution of Chilenia Terrane and Permo-Triassic tectonics of north Central Chile., Journal of South American Earth Sciences 47: 100-115,

Arévalo, C. 2005a. Carta Copiapó, Región de Atacama. Servicio Nacional de Geología y Minería, Carta Geológica de Chile, Serie Geología Básica, No. 91, 53 p., 1 mapa escala 1:100.000.

Arévalo, C., Creixell, C., 2009, The Atacama Fault System and its role on the formation of IOCG and Magnetite-Apatite ores: an evaluation from the Los Choros and Huasco valleys, Northern Chile, XII Congreso Geológico Chileno, Santiago, 22-26 Noviembre, 2009, S9_095.

Arévalo, C.; Creixell, C. 2010. Geología del área Cruz Grande-Junta de Chingoles, Región de Coquimbo. Servicio Nacional de Geología y Minería, Informe Registrado IR-10-40: 133 p., 6 mapas escala 1:50.000. Santiago.

Ávila, J.N., Chemale Jr, F., Mallmann, G., Borba, A.W. and Luft, F.F., 2005. Thermal evolution of inverted basins: Constraints from apatite fission track thermochronology in the Cuyo Basin, Argentine Precordillera. Radiation Measurements, 39(6), pp.603-611.

Barredo, S et al., 2012, Tectono-sequence stratigraphy and U–Pb zircon ages of the Rincón Blanco Depocenter, northern Cuyo Rift, Argentina, Gondwana Research, 49, 624-636

Bense, F.A., Löbens, S., Dunkl, I., Wemmer, K. and Siegesmund, S., 2013. Is the exhumation of the Sierras Pampeanas only related to Neogene flat-slab subduction? Implications from a multi-thermochronological approach. Journal of South American Earth Sciences, 48, pp.123-144.

Bissig, T., Clark, A.H., Lee, J.K.W., Heather, K.B., 2001. The Cenozoic history of volcanism and hydrothermal alteration in the Central Andean flat-slab region: New 40Ar-39Ar constrains from the El Indio-Pascua Au-Ag-Cu belt, 29°20-30°30" S. International Geology Review, 43, 312-340

Buelow, E.K., J. Suriano, J.B. Mahoney, D.L. Kimbrough, J.F. Mescua, L.B. Giambiagi, and G.D. Hoke, 2018, Sedimentologic and stratigraphic evolution of the Cacheuta basin: Constraints on the development of the Miocene retroarc foreland basin, south-central Andes, Lithosphere, 10, 366-391. GSA Data Repository Item 2018113L. DOI: https://doi.org/10.1130/L709.1

Capaldi, T.N., Horton, B.K., McKenzie, N.R., Mackaman-Lofland, C., Stockli, D.F., Ortiz, G. and Alvarado, P., 2020. Neogene retroarc foreland basin evolution, sediment provenance, and magmatism in response to flat slab subduction, western Argentina. Tectonics, 39(7), p.e2019TC005958.

Capaldi, T.N., McKenzie, N.R., Horton, B.K., Mackaman-Lofland, C., Colleps, C.L. and Stockli, D.F., 2021. Detrital zircon record of Phanerozoic magmatism in the southern Central Andes. Geosphere, 17(3), pp.876-897.

Casquet, C., Fanning, C.M., Galindo, C., Pankhurst, R.J., Rapela, C.W., Torres, P., 2010, The Arequipa Massif of Peru: New SHRIMP and isotope constraints on a Paleoproterozoic inlier in the Grenvillian orogen: Journal of South American Earth Sciences, v. 29, p. 128-142.

Castro de Machucal, Brigida, Lopez, Maria Gimena, Morata, Diego, 2015, La Aguadita Trachyte: new exponent of Triassic alkaline magmatism in Sierra de Valle Fertil, western Sierras Pampeanas, Revista de la Asociación Geológica Argentina 72, 551 - 562.

Cerda, A., 1984, Geologia del distrito minero Estero Quelen, Comuna de Salamanca, IV Region, Universidad de Chile, Departmento de Geologia y Geofisica, Memoria de Titulo, Inedito), 142 p., Santiago.

Collo, Gilda, Astini, Ricardo A., Cardona, Agustín, Do Campo, Margarita D., Cordani, Umberto, 2008, Edades de metamorfismo en las unidades con bajo grado de la región central del Famatina: la impronta del ciclo orogénico oclóyico (Ordovícico), Revista Geológica de Chile 35 (2): 191-213.

Coloma, F., Salazar, E., Creixell, C., 2012. Nuevos antecedentes acerca de la construcción de los plutones Pérmicos y Permo-Triásicos en el valle del río Tránsito, región de Atacama, Chile. In: XIII. Congreso Geológico Chileno Abstract S3_023, Antofagasta

Coloma, Felipe, Ximena Valin, Verónica Oliveros, Paulina Vásquez, Christian Creixell, Esteban Salazar, Mihai, N. Ducea, 2017, Geochemistry of Permian to Triassic igneous rocks

from northern Chile (28º-30º15'S): Implications on the dynamics of the proto-Andean margin, Andean Geology, 44, 147-178, doi: 10.5027/andgeoV44n2-a03

Colombo, F., C.O. Limarino, L.A. Spalletti, P. Busquets, R. Cardó, I. Méndez-Bedia, N. Heredia, 2014, Late Palaeozoic lithostratigraphy of the Andean Precordillera revisited (San Juan Province, Argentina), Journal of Iberian Geology 40 (2) 2014: 241-259, http://dx.doi.org/10.5209/rev_JIGE.2014.v40.n2.45311

Coughlin, T.J., O'Sullivan, P.B., Kohn, B.P. and Holcombe, R.J., 1998. Apatite fission-track thermochronology of the Sierras Pampeanas, central western Argentina: Implications for the mechanism of plateau uplift in the Andes. Geology, 26(11), pp.999-1002.

Creixell, C., Labbé, M., Arévalo, C., Salazar, E., 2013., Carta Geológica de Chile, Serie Geología Básica 150 Geología del área Estación Chañar-Junta de Chingoles, Regiones de Atacama y Coquimbo. escala 1:100000

Creixell, C., Ortiz, M., Arévalo, C., 2012, Geología del área Carrizalillo-El Tofo, Regiones de Atacama y Coquimbo., Servicio Nacional de Geología y Minería, Carta Geológica de Chile, Serie Geología Básica 133-134: 82 p., 1 mapa escala 1:100, 000, M-201,

Creixell, Christian, Javier Fuentes, Hessel Bierma, Esteban Salazar, 2015, Tectonic setting of Cretaceous porphyry belts of north Chile (28°-30° S), Conference: XIV Congreso Geologico Chileno, La Serena, Volume: AT2 ST5, 4p.

Cuitino_1985 Cuitino, L, 1985, Estudio geologico del yacimiento de lapislazuli Flor de los Andes, Memoria de Titulo. Univ. Chile, Depto. Geol. y Geofix., 120 p. Santiago

Dahlquist JA, Rapela CW, Pankhurst RJ, Fanning CM, Vervoort JD, Hart G, Baldo EG, Murra JA, Alasino PH, Colombo F (2012) Age and magmatic evolution of the Famatinian granitic rocks of sierra de Ancasti, Sierras Pampeanas, NW Argentina. J S Am Earth Sci 34:10–25

Dahlquist, J.A., Pankhurst, R., Rapela, C.W., Galindo, C., Alasino, P., Fanning, C.M., Saavedra, J., and Baldo, E., 2008, New SHRIMP U-Pb data from the Famatina complex: constraining early-mid Ordovician Famatinian magmatism in the Sierras Pampeanas, Argentina: Geologica acta: an international earth science journal, v. 6, p. 319-333.

Dahlquist, Juan A., Verdecchia, Sebastián O., Baldo, Edgardo G., Base,Miguel A.S., Alasino, Pablo H., Urán, Gimena A., Rapela, Carlos W., Campos Neto, Mario da Costa, and Zandomeni, Priscila S., 2016, Early Cambrian U-Pb zircon age and Hf-isotope data from the Guasayán pluton, Sierras Pampeanas, Argentina: implications for the northwestern boundary of the Pampean arc, Andean Geology 43 (1): 137-150.

Demant, Alain, Suárez, Manuel, De la Cruz, Rita, 2007, Geochronology and petrochemistry of Late Cretaceous-(?) Paleogene volcanic sequences from the eastern central Patagonian Cordillera (45°-45°40'S), Geocronología y petroquímica de sucesiones volcánicas del Cretácico Superior-¿Paleógeno? del este de la Cordillera Patagónica oriental (45°15□-45°40□S), Revista geológica de Chile, 34, 3-21, http://dx.doi.org/10.4067/S0716-02082007000100001

Drake, Robert E., Garniss Curtis, Mario Vergara, 1976b, Potassium-argon dating of igneous activity in the central Chilean Andes — latitude 33°S, Journal of Volcanology and Geothermal Research 1(3):285-295

Drobe, M., Lopez de Luchi, M.G., Steenken, A., Wemmer, K., Naumann, R., Frei, R. and Siegesmund, S., 2011. Geodymanic evolution of the Eastern Sierras Pampeanas (central Argentina) based on geochemical, Sm-Nd, Pb-Pb and SHRIMP data. Int. J. Earth Sci., 100, 631–657.

Ducea, Mihai N., Juan E. Otamendi, George Bergantz, Kelley M. Stair, Victor A. Valencia, and George E. Gehrels, 2010, Timing constraints on building an intermediate plutonic arc crustal section: U- Pb zircon geochronology of the Sierra Valle Fértil–La Huerta, Famatinian arc, Argentina, TECTONICS, VOL. 29, TC4002, doi:10.1029/2009TC002615.

Emparan, C., Pineda, G. 2000. Área La Serena-LaHiguera, Región de Coquimbo. Servicio Naciónal de Geología y Minería, Mapas Geológicos 18, escala 1:100.000.

Emparan, C., Pineda, G. 2006. Geología del Área Andacollo-Puerto Aldea, Región de Coquimbo. Servicio Nacional de Geología y Minería, Carta Geológica de Chile, Serie Geología Básica, No. 96, 85 p., 1 mapa escala 1:100.000.

Enkelmann, E et al., 2014, A thermochronometric view into an ancient landscape: Tectonic setting, development, and inversion of the Paleozoic eastern Paganzo basin, Argentina., Lithosphere, 128, 93-107

Espinoza, F., Matthews, S., Cornejo, P., Venegas, C., 2011, Carta Catalina, Región de Antofagasta., Servicio Nacional de Geología y Minería, Carta Geológica de Chile, Serie Geología Básica 129: 63 p., 1 mapa escala 1:100, 000, Santiago.,

Farrar E, Noble DC, 1976, Timing of late Tertiary deformation in the Andes of Peru. Geol. SocAmerica, Bull 87: 1247-1250

Fosdick, J.C., Carrapa, B. and Ortíz, G., 2015. Faulting and erosion in the Argentine Precordillera during changes in subduction regime: Reconciling bedrock cooling and detrital records. Earth and Planetary Science Letters, 432, pp.73-83.

Fox, K. 2000. Fe-oxide, Cu-U-Au-REE, mineralization and alteration at the Productora prospect. M.Sc. Thesis, Unpublished), Colorado School of Mines: 141 p.

Fuentes, Francisco, Vergara, Mario, Aquirre, Luis, Feraud, Gilbert, 2002, Relaciones de contacto de unidades volcanicas terciarias de los Andes de Chile central (33 S): una reinterpretacion sobre la base de dataciones 40Ar/39Ar, Revista Geologica de Chile, 29, 207-225.

"Fuentes, Francisco,Aguirre, Luis,Vergara, Mario,Valdebenito, Leticia,Fonseca, Eugenia,2004,Miocene fossil hydrothermal system associated with a volcanic complex in the Andes of central Chile,Journal of Volcanology and Geothermal Research,138,139-161.

"

GANA P., WALL R. M.: 40AR/39AR AND K-AR GEOCHRONOLOGICAL EVIDENCES OF AN UPPER CRETACEOUS-EOCENE HIATUS IN CENTRAL CHILE (33-33.5° S) REV. GEOL. CHILE 24 [1997] 145-163

Gana, P. 1991a. Mapa geológico de la Cordillera de la Costa entre La Serena y Quebrada El Teniente. Servicio Nacional de Geología y Minería, Documentos de Trabajo, No. 3, 1 mapa escala 1:100.000. Santiago.

Gibson S. A.; Thompson R. N.; Day J. A., 2006, Timescales And Mechanisms Of PlumeLithosphere Interactions: 40ar/39ar Geochronology And Geochemistry Of Alkaline Igneous Rocks From The Paranã ETENDEKA Large Igneous Province, Earth Planet. Sci. Lett.

Gibson S. A.; Thompson R. N.; Day J. A.; Humphris S. E.; Dickin A. P., 2005, MeltGeneration Processes Associated With The Tristan Mantle Plume: Constraints On The Origin Of Em1, Earth Planet. Sci. Lett.

Goddard, A.L.S. and Carrapa, B., 2018. Using basin thermal history to evaluate the role of Miocene–Pliocene flat-slab subduction in the southern Central Andes (27° S–30° S). Basin Research, 30(3), pp.564-585.

Godoy, E., 2012, Mapas geológicos preliminares de los cuadrángulos Carrizalillo e Incaguasi, regiones de Atacama y aCoquimbo., Servicio Nacional de Geología y Minería, Informe, Inédito), 2 mapas escala 1:50, 000,

Gordillo, C.E. ; Linares, E.. Geocronología and Petrografía of las vulcanitas terciarias del departamento Pocho, Provincia of Córdoba [artículos of revistas]. 1981. Publicado en: Asociación Geológica Argentina. Revista, 36 (4), pp. 380-388

Gulbranson, E.L., I.P. Montañez, M.D. Schmitz, C.O. Limarino, J.L. Isbell, S.A. Marenssi, and J.L. Crowley, 2010, High-precision U-Pb calibration of Carboniferous glaciation and climate

history, Paganzo Group, NW Argentina, Geological Society of America Bulletin; September/October v. 122; no. 9/10; p. 1480–1498; doi: 10.1130/B30025.1

Gulbranson, Erik L., Montañez, Isabel P., Tabor, Neil J., Oscar Limarino, C., 2015, Late Pennsylvanian aridification on the southwestern margin of Gondwana (Paganzo Basin, NW Argentina): A regional expression of a global climate perturbation, Palaeogeography, Palaeoclimatology, Palaeoecology, 417, 220-235.

Hervé, F., Calderón, M., Fanning, C.M., Pankhurst, R.J., and Godoy, E., 2013, Provenance variations in the Late Paleozoic accretionary complex of central Chile as indicated by detrital zircons: Gondwana Research, v. 23, p. 1122-1135.

Hervé, Francisco, C., Mark Fanning, Mauricio Calderón, Constantino Mpodozis, 2014, Early Permian to Late Triassic batholiths of the Chilean Frontal Cordillera, 28°–31°S): SHRIMP U–Pb zircon ages and Lu–Hf and O isotope systematics, Lithos 184–187, 436–446,

Hoke, G.D., Graber, N.R., Mescua, J.F., Giambiagi, L.B., Fitzgerald, P.G. and Metcalf, J.R., 2015. Near pure surface uplift of the Argentine Frontal Cordillera: insights from (U–Th)/He thermochronometry and geomorphic analysis.

Iannizzotto, Noelia F.. Carlos W. Rapela, Edgardo G. A. Baldo, Carmen Galindo, C.M. Fanning, Robert J. Pankhurst, 2013, The Sierra Norte-Ambargasta Batholith: Late Ediacaran-Early Cambrian magmatism associated with Pampean Transpressional Tectonics, Journal of South American Earth Sciences 42:127-143, DOI: 10.1016/j.jsames.2012.07.009

Jara, Pamela, Charrier, Reynaldo, 2014, Nuevos antecedentes estratigráficos y geocronológicos para el Meso-Cenozoico de la cordillera Principal de Chile entre 32° y 32°30’S: implicancias estructurales y paleogeográficas, Andean Geology 41, 174-209,

Jones, R., Kirstein, L., Kasemann, S., Dhuime, B., Elliott, T., Litvak, V., Alonso, R., y Edinburgh Ion Microprobe Facility (EIMF), 2015. Geodynamic controls on the contamination of Cenozoic arc magmas in the southern Central Andes: Insights from the O and Hf isotopic composition of zircon. Geochimica et Cosmochimica Acta 164: 386-402

Jones, R.E., Kirstein, L.A., Kasemann, S.A., Dhuime, B., Elliott, T., Litvak, V.D., Alonso, R., Hinton, R., 2015. Geodynamic controls on the contamination of Cenozoic arc magmas in the southern Central Andes: insights from the O and Hf isotopic composition of zircon. Geochimica et Cosmochimica Acta, 164, 386–402.

"Jones, Rosemary E., Kirstein, Linda A., Kasemann, Simone A., Litvak, Vanesa D., Poma, Stella, Alonso, Ricardo N., Hinton, Richard, EIMF, 2016, The role of changing geodynamics

in the progressive contamination of Late Cretaceous to Late Miocene arc magmas in the southern Central Andes, Lithos, 262, 169-191.

Jones, Rosemary E., Kirstein, Linda A., Kasemann, Simone A., Litvak, Vanesa D., Poma, Stella, Alonso, Ricardo N., Hinton, Richard, EIMF, 2016, The role of changing geodynamics in the progressive contamination of Late Cretaceous to Late Miocene arc magmas in the southern Central Andes, Lithos, 262, 169-191.

Jones, Rosemary E., Kirstein, Linda A., Kasemann, Simone A., Litvak, Vanesa D., Poma, Stella, Alonso, Ricardo N., Hinton, Richard, EIMF, 2016, The role of changing geodynamics in the progressive contamination of Late Cretaceous to Late Miocene arc magmas in the southern Central Andes, Lithos, 262, 169-191.

Jones, Rosemary E., Kirstein, Linda A., Kasemann, Simone A., Litvak, Vanesa D., Poma, Stella, Alonso, Ricardo N., Hinton, Richard, EIMF, 2016, The role of changing geodynamics in the progressive contamination of Late Cretaceous to Late Miocene arc magmas in the southern Central Andes, Lithos, 262, 169-191.

Jones, Rosemary E., Kirstein, Linda A., Kasemann, Simone A., Litvak, Vanesa D., Poma, Stella, Alonso, Ricardo N., Hinton, Richard, EIMF, 2016, The role of changing geodynamics in the progressive contamination of Late Cretaceous to Late Miocene arc magmas in the southern Central Andes, Lithos, 262, 169-191."

Jordan, T. E., Tamm, V., Figueroa, G., Flemings, P. B., Richards, D., Tabbutt, K., & Cheatham, T. (1996). Development of the MioceneManantiales foreland basin, Principal Cordillera, San Juan, Argentina. Andean Geology, 23(1), 43–79

Jordan, T.E., Allmendinger, R.W., Damanti, J.F. and Drake, R.E., 1993. Chronology of motion in a complete thrust belt: the Precordillera, 30-31 S, Andes Mountains. The Journal of Geology, 101(2), pp.135-156.

Jordan, T.E., Schlunegger, F. and Cardozo, N., 2001. Unsteady and spatially variable evolution of the Neogene Andean Bermejo foreland basin, Argentina. Journal of South American Earth Sciences, 14(7), pp.775-798.

Kay, S.M. and Abbruzzi, J.M., 1996. Magmatic evidence for Neogene lithospheric evolution of the central Andean “flat-slab” between 30 S and 32 S. Tectonophysics, 259(1-3), pp.15-28.

Kay, S.M. and Gordillo, C.E., 1994. Pocho volcanic rocks and the melting of depleted continental lithosphere above a shallowly dipping subduction zone in the central Andes. Contributions to Mineralogy and Petrology, 117(1), pp.25-44.

Kay, S.M., Mpodozis, C., Ramos, V.A. and Munizaga, F., 1991. Magma source variations for mid–late Tertiary magmatic rocks associated with a shallowing subduction zone and a thickening crust in the central Andes (28 to 33 S).

Kay, S.M., Mpodozis C, Tittler A, Cornejo P, 1996, Magmatic and Tectonic Controls on late Tertiary, Mineralization in the Maricunga Belt, III:117-122

Kay, S. M., Maksaev, V. A., Moscoso, R., Mpodozis, C., Nasi, C., 1987, Probing the evolving Andean lithosphere: Mid-Late Tertiary Magmatism in Chile (29°-30°30'S) over the modern zone of subhorizontal subduction. Journal of Geophysical Research, 92(B7), 6173-6189.

Kay, S.M., Mpodozis, C., and Coira B., 1999, Neogene magmatismo, tectonism and mineral deposit of the Central Andes (22° to 33°S Latitude). In Skinner B.J., ed., Geology and Ore Deposit of the Central Andes: Society Economic Geology Special Publ

Kay, S.M., Coira, Beatriz, Wörner, G.,Kay, Robert W., Singer, Brad, 2011, Geochemical, isotopic and single crystal 40Ar/39Ar age constraints on the evolution of the Cerro Galán ignimbrites, Bull Volcanol, 2011, 73: 1487. https://doi.org/10.1007/s00445-010-0410-7

Leveratto,M. A. 1976. Edad of intrusives cenozoicos en la Precordillera of San Juan and su implicancia estratigrafica. Revista of la Asociacio ́n Geolo ́gica Argentina, 31, 53-58

Levina, M et al., 2014, Cenozoic sedimentation and exhumation of the foreland basin system preserved in the Precordillera thrust belt (31-32°S), southern central Andes, Argentina, Tectonics, 485, 1659-1680

Limarino, C.O., Gutiérrez, P. R., Malizia, D., Barreda, V., Page, S., Ostera, H. y Linares, E., 1999. Edad de las secuencias paleógenas y neógenas de las cordilleras de la Brea y Zancarrón, Valle del Cura, San Juan. Revista de la Asociación Geológica Argentina 54(2): 177-181.

Litvak, V. y Poma, S. 2014. Petrogenesis of Miocene volcanic arc rocks over the Chilean Pampean flat-slab segment of the Central Andes constrained by mineral chemistry. Geologica Acta 12:151-170.

Litvak, V.D. 2009. El volcanismo Oligoceno superior – Mioceno inferior del Grupo Doña Ana en la Alta Cordillera de San Juan. Revista de la Asociación Geológica Argentina 64: 201-213

Litvak, V.D., Kay, S.M. y Mpodozis, C. 2005b. New K/Ar ages on Tertiary Volcanic Rocks in the Valle del Cura, Pampean flat slab segment, Argentina. 16º Congreso Geológico Argentino, Actas 2: 159-164, La Plata.

Litvak, V.D., Poma, S. and Kay, S.M., 2007. Paleogene and Neogene magmatism in the Valle del Cura region: new perspective on the evolution of the Pampean flat slab, San Juan province, Argentina. Journal of South American Earth Sciences, 24(2-4), pp.117-137.

Litvak, V.D., Poma, S., Jones, R.E., Fernández Paz, L., Iannelli, S.B., Spagnuolo, M., Kirstein, L.A., Folguera, A. and Ramos, V.A., 2018. The late Paleogene to Neogene volcanic arc in the southern Central Andes (28–37 S). The Evolution of the Chilean-Argentinean Andes, pp.503-536.

Litvak, V.D., Poma, S., Kay, S.M., 2007. Paleogene and Neogene magmatism in the Valle del Cura region: a new perspective on the evolution of the Pampean flat slab, San Juan province, Argentina. Journal of South American Earth Science, 24(2-4), 117-137.

Litvak, Vanesa D., y Stella Poma, 2005a, Estratigrafía y facies volcánicas y volcaniclásticas de la Formación Valle del Cura: magmatismo paleógeno en la Cordillera Frontal de San Juan, Revista de la Asociación Geológica Argentina, 60 (2): 402-416.

Löbens, S., Bense, F.A., Dunkl, I., Wemmer, K., Kley, J. and Siegesmund, S., 2013. Thermochronological constraints of the exhumation and uplift of the Sierra de Pie de Palo, NW Argentina. Journal of South American Earth Sciences, 48, pp.209-219.

Lossada, A.C., Giambiagi, L., Hoke, G.D., Fitzgerald, P.G., Creixell, C., Murillo, I., Mardonez, D., Velásquez, R. and Suriano, J., 2017. Thermochronologic evidence for late Eocene Andean mountain building at 30 S. Tectonics, 36(11), pp.2693-2713.

Lucassen F.; Franz G.; Romer R. L.; Schultz F.; Dulski P.; Wemmer K., 2007, PreCenozoic IntraPlate Magmatism Along The Central Andes (1734Â°S): Composiiton Of Thze Mantle At An Active Margin, Lithos

Mackaman-Lofland, C., Horton, B.K., Fuentes, F., Constenius, K.N., Ketcham, R.A., Capaldi, T.N., Stockli, D.F., Ammirati, J.B., Alvarado, P. and Orozco, P., 2020. Andean mountain building and foreland basin evolution during thin-and thick-skinned Neogene deformation (32–33° S). Tectonics, 39(3), p.e2019TC005838.

Mackaman-Lofland, C., Horton, B.K., Ketcham, R.A., McQuarrie, N., Fosdick, J.C., Fuentes, F., Constenius, K.N., Capaldi, T.N., Stockli, D.F. and Alvarado, P., 2022. Causes of variable shortening and tectonic subsidence during changes in subduction: Insights from flexural thermokinematic modeling of the Neogene southern central Andes (28–30 S). Tectonics, 41(8), p.e2022TC007334.

Mackaman-Lofland, C., Lossada, A.C., Fosdick, J.C., Litvak, V.D., Rodríguez, M.P., del Llano, M.B., Ketcham, R.A., Stockli, D.F., Horton, B.K., Mescua, J. and Suriano, J., 2024. Unraveling the tectonic evolution of the Andean hinterland (Argentina and Chile, 30° S)

using multi-sample thermal history models. Earth and Planetary Science Letters, 643, p.118888.

Mackaman-Loflanda, Chelsea, Brian K.Horton, Facundo Fuentes, Kurt N. Constenius, Daniel F.Stockli, 2019, Mesozoic to Cenozoic retroarc basin evolution during changes in tectonic regime, southern Central Andes (31–33°S): Insights from zircon U-Pb geochronology, Journal of South American Earth Sciences, 89, 2019, 299-318.

Maksaev, Víctor, Francisco Munizaga1, Colombo Tassinari, Timing of the magmatism of the paleo-Pacific border of Gondwana: U-Pb geochronology of Late Paleozoic to Early Mesozoic igneous rocks of the north Chilean Andes between 20° and 31°S, 2014, Andean Geology 41, 447-506.

Maksaev, Víctor, Tomás A., Almonacid, Francisco Munizaga, Víctor Valencia, Michael McWilliams, Fernando Barra, 2010, Geochronological and thermochronological constraints on porphyry copper mineralization in the Domeyko alteration zone, northern Chile, Andean Geology 37: 144-176,

Mancuso, AC et al., 2010, Age constraints for the northernmost outcrops of the Triassic Cuyana Basin, Argentina, J South Amer Earth Sci, PhD Disser 3496, 97-103

Mardonez, D., Suriano, J., Giambiagi, L., Mescua, J., Lossada, A., Creixell, C. and Murillo, I., 2020. The Jáchal river cross-section revisited (Andes of Argentina, 30 S): Constraints from the chronology and geometry of neogene synorogenic deposits. Journal of South American Earth Sciences, 104, p.102838.

Martin, M. W. 1996. Report on U-Pb geochronology conducted at the Isotope Geochemistry Laboratory, University of Kansas, between June 17 and August 30, 1996 on samples collected for the Decima Norte project. Servicio Nacional de Geología y Minería. Informe inédito. 41p.

Martin, M.W., Clavero, J., Mpodozis, C. y Cuitiño, L. 1995. Estudio Geológico de la Franja El Indio, Cordillera de Coquimbo: Servicio Nacional de Geología y Minería, Informe Registrado IR-95-6, 1: 1-238, Santiago

Martina, Federico, Horacio Nicolas Canelo, Federico M. Dávila, María Helena M. de Hollanda, Wilson Teixeira, 2018, Mississippian lamprophyre dikes in western Sierras Pampeanas,Argentina: Evidence of transtensional tectonics along the SW margin of Gondwana, Journal of South American Earth Sciences 83, 68-80.

MARTINEZ ARDILA A. M., CLAUSEN B. L., MEMETI V., PATERSON S. R.: SOURCE CONTAMINATION, CRUSTAL ASSIMILATION, AND MAGMATIC RECYCLING DURING THREE FLARE-UP EVENTS IN THE CRETACEOUS PERUVIAN COASTAL BATHOLITH: AN EXAMPLE

FROM THE ICA-PISCO PLUTONS J. S. AMER. EARTH SCI. 95 (102300) [2019] doi: 10.1016/j.jsames.2019.102300

Maydagán, L., Franchini, M., Chiaradia, M., Pons, J., Impiccini, A., Toohey, J., and Rey, R., 2011, Petrology of the Miocene igneous rocks in the Altar region, main cordillera of San Juan, Argentina., A geodynamic model within the context of the andean flat-slab segment and metallogenesis: Journal of South American Earth Sciences, v., 32, p., 30–48,

Maydagán, L., Zattin, M., Mpodozis, C., Selby, D., Franchini, M. and Dimieri, L., 2020. Apatite (U–Th)/He thermochronology and Re–Os ages in the Altar region, Central Andes (31° 30' S), Main Cordillera of San Juan, Argentina: implications of rapid exhumation in the porphyry Cu (Au) metal endowment and regional tectonics. Mineralium Deposita, 55, pp.1365-1384.

Maydagán, Laura, Marta Franchini, Massimo chiaradia, John Dilles, and Roger Rey, 2014, The Altar Porphyry Cu-(Au-Mo, Deposit, Argentina): A Complex Magmatic-Hydrothermal System with Evidence of Recharge Processes, Economic Geology, v., 109, pp., 621–641

Milana, J.P., Bercowski, F. and Jordan, T., 2003. Paleoambientes y magnetoestratigrafía del Neógeno de la Sierra de Mogna, y su relación con la Cuenca de Antepaís Andina. Revista de la Asociación Geológica Argentina, 58(3), pp.447-473.

Molina, Pablo G., Miguel A. Parada, Francisco J. Gutiérrez, Changqian Ma, Jianwei Li, Liu Yuanyuan, Martin Reich, Álvaro Aravena, 2015, Protracted late magmatic stage of the Caleu pluton (central Chile) as a consequence of heat redistribution by diking: Insights from zircon data and thermal modeling, Lithos,Volume 227, 255-268.

Montecinos, P., 1983, Petrologie des roches intrusive associees au gisement de for El Algarrobo (Chili). These de docteur, Universite de Paris-Sud, Centre d'Orsay, 326 p. Paris, France.

Montecinos, P., Schärer, U., Vergara, M., and Aguirre, L., 2008, Lithospheric origin of, Oligocene-Miocene magmatism in Central Chile: U-Pb ages and Sr-Pb-Hf isotope, composition of minerals: Journal of Petrology, 49, 555-580.

Montecinos, P., Schärer, U., Vergara, M., and Aguirre, L., 2008, Lithospheric origin of, Oligocene-Miocene magmatism in Central Chile: U-Pb ages and Sr-Pb-Hf isotope, composition of minerals: Journal of Petrology, 49, 555-580.

Morata, D et al., 2010, Peraluminous Grenvillian TTG in the Sierra de Pie de Palo, Western Sierras Pampeanas, Argentina: Petrology, geochronology, geochemistry and petrogenetic implications, Precambrian Research, 177, 308-322.

Moscoso, R., Mpodozis, C., Nasi, C., Ribba, L., Arévalo, C. 2010. Geología de la Hoja El Tránsito, Región de Atacama, Mapa de compilación 1:250.000, Servicio Nacional de Nº 7, Santiago.Geología y Minería, Carta Geológica de Chile, Serie Preliminar

Mpodozis C, Kay S, Gardeweg M, Coira B, editors. Geología de la región de Ojos del Salado (Andes centrales, 27 S): implicancias de la migración hacia el este del frente volcánico Cenozoico Superior. Congreso Geológico Argentino; 1996.

Mpodozis, C., Cornejo, P. 1988. Hoja Pisco Elqui, IV Región de Coquimbo. Servicio Nacional de Geología y Minería, Carta Geológica de Chile, No. 68, 164 p.

Mpodozis, Constantino, and Paula Cornejo, 2012, Cenozoic tectonics and porphyry copper systems of the Chilean Andes, 2012, SOCIETY OF ECONOMIC GEOLOGISTS, INC., Special Publication Number 16, J.W. Hedenquist, M. Harris, and F. Camus, Editors, Chapter 14, 329-361.

Mpodozis, Constantino, and Paula Cornejo, 2012, Cenozoic tectonics and porphyry copper systems of the Chilean Andes, 2012, SOCIETY OF ECONOMIC GEOLOGISTS, INC., Special Publication Number 16, J.W. Hedenquist, M. Harris, and F. Camus, Editors, Chapter 14, 329-361.

Munizaga V., Francisco, Vicente, Jean Claude, 1982, Acerca de la zonacion plutonica y del volcanismo miocenico en los Andes de Aconcagua, lat. 32-33°S : datos radiometricos KAr. -Palabra Clave

Munizaga, F., 1972, Edades radiometricas de rocas chilenas, In Inst Invest. Geol., Jornadas de Trabajo, 2, 1, 132-145. Antofagasta.

Munizaga, Francisco, Victor Maksaev, C., M., Fanning, S., Giglio, G., Yaxley, and C., C., G., Tassinari, 2008, Late Paleozoic–Early Triassic magmatism on the western margin of Gondwana: Collahuasi area, Northern Chile, Gondwana Research, 13, Issue 3, April 407-427,

Ortiz, G., Alvarado, P., Fosdick, J.C., Perucca, L., Saez, M. and Venerdini, A., 2015. Active deformation in the northern sierra de Valle Fértil, sierras Pampeanas, Argentina. Journal of South American Earth Sciences, 64, pp.339-350.

Ortiz, G., Goddard, A.L.S., Fosdick, J.C., Alvarado, P., Carrapa, B. and Cristofolini, E., 2021. Fault reactivation in the Sierras Pampeanas resolved across Andean extensional and compressional regimes using thermochronologic modeling. Journal of South American Earth Sciences, 112, p.103533.

Ortiz, M., Merino, R.N. Geología de las áreas Río Chollay - Matancilla y Cajón del Encierro, Regiones de Atacama y Coquimbo. Escala 1:100.000.Carta Geológica de Chile, Serie Geología Básica. Nos. 175-176.

Pankhurst, R., Millar, I., Hervé, F., 1996, A Permo-Carboniferous U-Pb age for part of the Guanta unit of the Elqui-Limarí Batholith at Río del Tránsito, Northern Chile: Revista Geológica de Chile 23: 35-42,

Pankhurst, R.J., Riley, T.R., Fanning, C.M., Kelley, S.P., 2000, Episodic silicic volcanism in Patagonia and the Antarctic Peninsula: chronology of magmatism associated with the breakup of Gondwana, J. Petrol., 41, 603-625

Parada Velazquez, F.N., 2013. Geoquímica of las rocas ígneas del Carbonífero-Trásico of la Alta Cordillera, Region of Atacama, Chile. Memoria para optar a Título of Geólogo, Santiago of Chile, 94 pp.

Parada, M.A., Féraud, G., Fuentes, F., Aguirre, L., Morata, D., and Larrondo, P., 2005, Ages and cooling history of the Early Cretaceous Caleu pluton: testimony of a switch from a rifted to a compressional continental margin in central Chile: Journal of the Geological Society, v. 162, p. 273-287.

Peña, M., 2012, Reconocimiento del límite sur del patrón paleógeno de rotaciones horarias entre los 28º-32ºS del margen chileno a través de un estudio paleomagnético., Memoria de Titulo, Universidad de Chile, Departamento de Geología: 81 p.,

Perelló, J., Urzœa, F., Cabello, J., and Ortiz, F., 1996, Clustered, gold-bearing Oligocene porphyry copper and associated epithermal mineralization at La Fortuna, Vallenar region, northern Chile, in Camus, F., Sillitoe, R.H., and Petersen, R., eds., Andean copper deposits: new discoveries, mineralization, styles and metallogeny: Society of Economic Geologists Special Publication no. 5, p. 81-90.

Perelló, Jose, Richard H. Sillitoe, Mpodozis, C., Humberto Brockway, AND Hector Posso, 2012, Geologic Setting and Evolution of the Porphyry Copper-Molybdenum and Copper-Gold Deposits at Los Pelambres, Central Chile, Chapter 4, Society of Economic Geologists, Inc., Special Publication 16, 79–104.

Pineda, G., Calderón, M., 2008, Hoja Monte Patria-El Maqui, Región de Coquimbo., Servicio Nacional de Geología y Minería, Carta Geológica de Chile, Serie Geología Básica 116: 44 p., 1 mapa escala 1:100, 000, Santiago.,

Pineda, G.,,Emparan, C., 2006, Geología del área Vicuña-Pichasca, Región de Coquimbo., Servicio Nacional de Geología y Minería, Carta Geológica de Chile, Serie Geología Básica, No., 97, 40 p., 1 mapa escala 1:100, 000, Santiago.,

Pineda, German, Emparan, Carlos, 1997, Nuevos antecedentes de la estratigraphia y geocronologiz Cretacica del norte del Rio Elqui: evidencias de tectonica extensional, VIII Congreso Geologico Chileno, Departameto de Ciencias Geologicas, Universidad Catolica del Norte, 215-219.

Pinto, L., Alarcón, P., Morton, A., & Naipauer, M. (2018). Geochemistry of heavy minerals and U–Pb detrital zircon geochronology in theManantiales Basin: Implications for Frontal Cordillera uplift and foreland basin connectivity in the Andes of central Argentina.Palaeogeography, Palaeoclimatology, Palaeoecology, 492, 104–125

Piquer, J., Hollings, P., Rivera, O., Cooke, D.R., Baker, M. and Testa, F., 2017. Along-strike segmentation of the Abanico Basin, central Chile: New chronological, geochemical and structural constraints. Lithos, 268, pp.174-197.

Plonka, Z.C., Capaldi, T.N., Odlum, M.L., Mackaman-Lofland, C., Ortiz, G. and Alvarado, P., 2023. Along-strike tectonic evolution of the Neogene Bermejo foreland basin and Eastern Precordillera thrust front, Argentina (30-32° S). Journal of South American Earth Sciences, 129, p.104521.

Poma, Stella, Adriana Ramos, Vanesa D. Litvak, Sonia Quenardelle, Emma B. Maisonnave, Iris Díaz, 2017, Southern Central Andes Neogene magmatism over the Pampean Flat Slab: implications on crustal and slab melts contribution to magma generation in Precordillera, Western Argentina, Andean Geology 44 Andean Geology 44, 249-274, doi: 10.5027/andgeoV44n3-a02

Quirt, S., Clark, A. H., Farrar, E., 1971, Potassium-argon ages of porphyry copper deposits in northern and central Chile., Abs., Geological Society of America, Abs., 3, 7, 676-677.

Ramos V. A.; Kay S. M., 1991b, Triassic Rifting And Associated Basalts In The Cuyo Basin, Central Argentina, Spec. Pap. Geol. Soc. Am.

"Ramos V. and Folguera A., 2009, Andean flat-slab subduction through time. From: MURPHy, J. B., KEPPIE, J. D. & HyNES, A. J. (eds) Ancient Orogens and Modern Analogues.

Geological Society, London, Special Publications, 327, 31–54."

Ribba, L. 1985. Geología regional del cuadrángulo El Tránsito, Región de Atacama, Chile. Universidad de Chile, Departamento de Geología, Memoria de título: 203 p., 2 mapas fuera de texto. Santiago.

Ribba, L., Mpodozis M, C., Hervé, F., Nasi, C., Moscoso, R., 1988, El basamento del valle del Tránsito, Cordillera de Vallenar: Eventos magmáticos y metamórficos y su relación con la evolución de los Andes chileno-argentinos: Revista Geológica de Chile 15: 129-150,

Rivano G., Sergio, Sepulveda H., Patricio, Hervé A., Miguel, Puig G., Alvaro, 1985, Geocronologia KAr de las rocas intrusivas entre los 31 deg - 32 deg Latitude South, Chile, Revista Geologica de Chile, 24, 63-74.

Rivano, S., Sepulveda, P. 1991 Hoja Illapel, Region de Coquimbo, Serv. Nac. Geol. Miner., Carta Geol Chile.

Rocha-Campos, A.C., Basei, M.A., Nutman, A.P., Kleiman, L.E., Varela, R., Llambias, E., Llambias, E., Canile, F.M., and da Rosa, O., 2011, 30 million years of Permian volcanism recorded in the Choiyoi igneous province (W Argentina) and their source for younger ash fall deposits in the Paraná Basin: SHRIMP U-Pb zircon geochronology evidence: Gondwana Research, v. 19, p. 509-523.

Rodríguez, M.P., Charrier, R., Brichau, S., Carretier, S., Farías, M., de Parseval, P. and Ketcham, R.A., 2018. Latitudinal and longitudinal patterns of exhumation in the Andes of north-central Chile. Tectonics, 37(9), pp.2863-2886.

Rojas Martinez, Paula Amanda, 2017, Genesis of the Le Romeal Iron Ore: New contributions to the understanding of iron oxide-apatite deposits, Thesis paraj optar al Grado de Magister en Ciencias, Mencion Geologia Memoria para Optar al Titulo de Geologa, Departmento de Geologia, Facultad de Ciencias Fisicas y Matematicas, Universidad de Chile.

Romo, José Meulén Piquer, 2015, Structural Geology of the Andes of Central Chile: Controls on Magmatism and the Emplacement of Giant Ore Deposits, ARC Centre of Excellence in Ore Deposits, CODES), School of Physical Sciences, University of Tasmania, Australia

Ronemus, C.B., Howlett, C.J., DeCelles, P.G., Carrapa, B. and George, S.W., 2024. The Manantiales basin, southern Central Andes (~ 32 S), preserves a record of late Eocene–Miocene episodic growth of an east-vergent orogenic wedge. Tectonics, 43(3), p.e2023TC008100.

Rossel, P., Oliveros, V., Ducea, M., Charrier, R., Scaillef, S., Retamal, L., and, Figueroa, O., 2013, The Early Andean Subduction system as an analogue to island arcs: evidence froam across-arc geochemical variations in northern Chile., Lithos 3063,

Salazar, E., 2012, Evolución tectonoestratigráfica de la cordillera de Vallenar: Implicancias en la construcción del oroclino de Vallenar., Universidad de Chile, Departamento de Geología, Tesis de Magíster: 126 p., 1 mapa escala 1:100, 000, Santiago.,

Salazar, E., and Coloma, F., 2016, Geologia del area Cerros de Cantaritos-Laguna Chica, Region de Atacama, Servicio Natcional de Geologia y Mineria, Carta Geologica de Chile, Serie Geologia Basica 181:171 p., 1 mapa escala 1:100, 000,

Salazar, E., Coloma, F., and Creixell, C., 2013, Geología del área El Tránsito-Lagunillas, Región de Atacama, Servicio Nacional de Geología y Minería, Carta Geológica de Chile, Serie Geología Básica 149: 106 p., 1 mapa escala 1:100, 000,

Salazar, J., Sarmiento, J., Vidal, C.E., Noble, D.C., 2009, Bonanza gold-telluride epithermal vein mineralization of the Chipmo zone, Orcopampa district, southern Peru: Sociedad Geologica del Peru, Volumen Especial, n. 7, p. 107-149.

Sillitoe, Richard.H., 1977, Permo-Carboniferous Upper Cretaceous, and Miocene porphyry copper-type mineralization in the Argentinian Andes, Economic Geology, 72, 99-109.

Stalder, N.F., Herman, F., Fellin, M.G., Coutand, I., Aguilar, G., Reiners, P.W. and Fox, M., 2020. The relationships between tectonics, climate and exhumation in the Central Andes (18–36 S): Evidence from low-temperature thermochronology. Earth-Science Reviews, 210, p.103276.

Steenken, A., Siegesmund, S., Wemmer, K., and López de Luchi, M.G., 2008, Time constraints on the Famatinian and Achalian structural evolution of the basement of the Sierra de San Luis, Eastern Sierras Pampeanas, Argentina: Journal of South American Earth Sciences, v. 25, p. 336-358.

Suriano, J., Lossada, A.C., Mahoney, J.B., Tedesco, A.M., Limarino, C.O., Giambiagi, L.B., Mazzitelli, M.A., Mescua, J.F., Lothari, L. and Quiroga, R., 2023. The southern extension of the Eocene Andean orogeny: New sedimentary record of the foreland basin in the southern Central Andes at 32° S. Basin Research, 35(6), pp.2381-2400.

Tedesco, Ana, Carlos Oscar Limarino, Patricia L Ciccioli, 2007, Primera edad radimétrica de los depósitos Cretácicos de la Precordillera Central, Revista de la Asociacion Geologica Argentina 62, 471-474."

Thiele, R., y Hervé, F. 1984, Sedimentacion y tectonica de antearco en los terrenos preandinos del Norte Chico de Chile, Revista Geologica de Chile, 22, 61-75.

Toselli G. A., 1978, Age Of The Negro Peinado Formation, Sierra Del Famatina, La Rioja, Rev. Asoc. Geol. Argentina

Tsotsos, J., P., 2006, Getting to know the Chehueque Pluton: an in Depth Study of Emplacement using Geochronology., Undergraduate Research Project, Unpublished), University of Toronto: 43 p.,

Urbina, N., Sruoga, P., Malvicini, L., 1997, Lat Tertiary gold-bearing volcanic belt in the Sierras Pampeanas of San Luis, Argentina, International Geology Review 39, 287-306.

Valenzuela, J. 2002. Caracterización, geocronología y mecanismos de emplazamiento del Batolito de la Costa en Vallenar, 28°22´-28°41´S y 70°45´-71°7´W), III Región, Chile. Memoria de Título, Inédito), Universidad de Concepción,118 p. Concepción.

Vergara H. Cuadrángulo Ujina, Región de Tarapacá, Carta Geológica de Chile, Escala 1: 50,000. 1978.

Vergara, M., Charrier, R., Munizaga, A. F., Rivano, S., Sepulveda, P., Thiele, R., Drake, R, 1988, Miocene volcaism in the Central Chilean Andes, 31 deg 30'S-34 deg 35' S), Journal of South American Earth Sciences, 1, 2, 199-209.

Vergés, J., Ramos, E., Seward, D., Busquets, P. and Colombo, F., 2001. Miocene sedimentary and tectonic evolution of the Andean Precordillera at 31 S, Argentina. Journal of South American Earth Sciences, 14(7), pp.735-750.

Welkner, D., Arévalo, C., Godoy, E., 2006, Geología de la Carta Freirina-El Morado, Región de Atacama, Servicio Nacional de Geología y Minería, Carta Geológica de Chile, Serie Geología Básica, No., 100, 50 p., 1 mapa escala 1:100, 000,

Winocur et al. 2015 Winocur, D.A., Litvak, V. y Ramos, V.A. 2015. Magmatic and tectonic evolution of the Oligocene Valle del Cura basin, main Andes of Argentina and Chile: evidence for generalized extension. En Sepúlveda, S., Giambiagi, L., Pinto, L., Moreiras, S., Tunik, M., Hoke, G. y Farías, M. (eds.) Geodynamic Processes in the Andes of Central Chile and Argentina, Geological Society, Special Publications 399:109-130, London.

Winocur, D.A., Litvak, V.D. and Ramos, V.A., 2015. Magmatic and tectonic evolution of the Oligocene Valle del Cura basin, main Andes of Argentina and Chile: evidence for generalized extension.

Zentilli, M., 1974, Geological evolution and metallogenetic relationships in the Andes of northern Chile between 26° and 29°south., Ph., D., Thesis, unpublished), Queen's University, Kingston, p., 394, Canada.,
