# Supplementary material for: Inboard advance of arc magmatism regulates mountain building in the Andes
Source: Nat Commun. 2026 Apr 11;17:5082. doi: 10.1038/s41467-026-71431-x (PMC13247209; doi:10.1038/s41467-026-71431-x)
Supplement: Supplementary file 2 — Description of Additional Supplementary Files [file 41467_2026_71431_MOESM2_ESM.pdf]

Description of Additional Supplementary Files

Supplementary Data 1: Igneous Geochronology Date Compilation (Fig. 2A; Supplemental Figure 1)

Supplementary Data 2: Compilation of Bedrock Nd isotope data (Fig. 1B; Fig. 3B)

Supplementary Data 3: Compilation of Bedrock and Detrital Zircon Lu-Hf data (Fig. 1B; Fig. 3B)

Supplementary Data 4: Apatite (U-Th-Sm)/He Thermochronology Date Compilation (Fig. 2A; Supplemental Figure 2; Supplemental Figure 3)

Supplementary Data 5: Apatite Fission Track Thermochronology Date Compilation (Fig. 2A; Supplemental Figure 2)

Supplementary Data 6: Deformation Front constraints from synorogenic stratigraphic data, structural and cross-cutting relationships, thermochronology data, and thermokinematic modeling (Fig. 2A).

Supplementary Data 7: Chronostratigraphic data used for Retroarc foreland basin sediment accumulation histories (Fig. 3D). 1: Calingasta; 2: Talacasto; 3: Manantiales; 4: Albarracín; 5: proximal Bermejo (Sierra Villicum); 6: proximal Bermejo (Mogna Anticline); 7: distal Bermejo (Ampacama)
