# Supplementary material for: Inboard advance of arc magmatism regulates mountain building in the Andes
Source: Nat Commun. 2026 Apr 11;17:5082. doi: 10.1038/s41467-026-71431-x (PMC13247209; doi:10.1038/s41467-026-71431-x)
Supplement: Supplementary file 4 — Transparent Peer Review file [file 41467_2026_71431_MOESM4_ESM.pdf]

nature portfolio

Peer Review File

Inboard advance of arc magmatism regulates mountain building in the Andes

Corresponding Author: Dr Tomas Capaldi

This file contains all reviewer reports in order by version, followed by all author rebuttals in order by version.

Attachments originally included by the reviewers as part of their assessment can be found at the end of this file.

Version 0:

Reviewer comments:

Reviewer #1

(Remarks to the Author)
This contribution by Capaldi and coauthors integrates geo/thermochronological, geochemical and stratigraphic records of Cenozoic arc magmatism to shed light on the interplay between subduction dynamics, arc magmatism, back-arc crustal shortening and flexural subsidence in Andean-type subduction orogens. To achieve this, a compilation of igneous rock crystallization ages, combined with zircon U-Pb geochronology and εHf isotopic compositions, provides valuable insights into the mechanisms governing these complex geological processes.

The manuscript presents a compelling case for the role of short-term (5-8 Myr) tectonic-magma cycles in controlling deformation and foreland basin subsidence. This represents a significant advancement over previous models that have primarily focused on longer (10-20 Myr) episodes of magmatism, deformation and crustal thickening. The discussion on how these shorter cycles particularly affect regions with lower magnitudes of shortening is insightful and adds an important dimension to our understanding of Andean orogenesis.

Overall, this paper is well-framed, clear, concisely written and technically sound. However, I have a few suggestions that might enhance its clarity and impact:

• The timing of εHf isotopic variations could be more precisely constrained. For instance, in Figure 3, while the trend between 28 Ma and 22 Ma is well-explained, the period between 25-22 Ma shows both positive and negative εHf values, suggesting contributions from both depleted-mantle and enriched-crustal melts. A discussion of the possible causes in the text for this mixed signal would be beneficial.

• The second pull-down phase (19-13 Ma) is linked to the expansion of arc magmatism into the Precordillera. However, the isotopic pull-down appears to begin after 16-17 Ma. A clarification of this apparent discrepancy would be helpful.

• The subsidence of the Talacasto Basin, located far from the thrust front in the Main Cordillera, is intriguing. The absence of similar subsidence evidence in the Calingasta and Manantiales Basins at the eastern foot of the Frontal Cordillera also warrants further discussion.

• Figure 4: This is an excellent and self-explanatory figure. I suggest incorporating a time-referencing framework from the Southern Central Andes example to provide additional and specific context for different phases.

Additional points for consideration on this Figure:

• Why is there no lithospheric mantle beneath the arc axis in Step 1? I suggest changing the figure or explaining why not?

• In Step 2, could you elaborate on how fluids migrate to the retroarc region?

• In Step 3, does the model require a normal or thin crust? Could you expand on how flexural subsidence in the foreland relates to shortening?

Minor issues:

• Abstract: Clarify why magmatism migrates in your model and whether slab flattening is a necessary condition or not.

• Line 51: Further explain why landward arc magmatism migration enhances fluid flux.

• Lines 67-69: The phrase "spatial and temporal variations" could be more specific.

• Line 104: The study focuses on the relationship between arc magmatism and retroarc crustal shortening, rather than broad crustal deformation. Please correct.

• Lines 115-117: Address the proposed crustal extension during the late Oligocene-Early Miocene (25-20 Ma).

• Lines 119-129: Discuss the isotopic pull-up periods (21-18 Ma, 13-11 Ma, 7-6 Ma), which are visible in Figure 3 but not explained in the text.

• Lines 157-158: Clarify the timing of major subsidence and its correlation with the Main Cordillera shortening.

• Line 160: Define "high-angle reverse faults" more clearly.

• Lines 204-206: Could you specify which retroarc regions are weakened by slab-derived fluids?

• Line 210: The term "High-Flux Events (HFEs)" should be introduced earlier with an explanation.

• Figure 1: Add basin names to the caption.

Overall, this is a valuable contribution to the field. However, addressing the points above would further strengthen the manuscript and clarify key interpretations. I would strongly encourage publication, following these refinements.

Best regards,
Laura Giambiagi

Reviewer #2

(Remarks to the Author)
Dear Editor,

In this letter I send you my comments on the paper entitled “Inboard advance of arc magmatism regulates mountain building in the Andes” coauthored by Capaldi, T., Horton, B., Mackaman-Lofland, C., Fuentes, F. and Ortiz, G.

This work explores the interactions and feedbacks among subduction, magmatism, and crustal deformation in a continental margin setting taking as a case study the southern Central Andes, mostly between ~29°-33°S. It is based on an extensive compilation of data that includes igneous ages, apatite (U-Th-Sm)/He and fission track thermochronology, and zircon Hf isotopic composition, in addition to calculations of sediment accumulation rates and deformation front reconstructions also based on the information reported by previous studies. The main text of the manuscript is well organized, the 4 figures included are appropriate to illustrate the issues discussed, and there is an extensive supplementary material which provides the data on which the work is based. I do consider that the figures and the supplementary material could be improved (see below).

As a main result, the work proposes a cyclic model that links short-term orogenic phases with shifts in thermal and rheological processes with the following sequential events: (1) arc magmatism weakens the crust, (2) the latter can then be deformed mostly by retroarc shortening with westward underthrusting, (3) crustal thickening prompts slab shallowing and a consequent eastward arc migration, (4) the newly allocated arc starts the cycle again. The model is very appealing as it offers a linked explanation of major processes observed throughout Andean orogenic construction: the eastward migration of arc magmatism and of deformation. In addition, as stated in the manuscript itself, the model focuses on internal orogenic cycles much shorter (<10 Myr) and of less magnitude (<50-150 km horizontal shortening) than those already covered in previous works (>20-50 Ma and >200-300 km horizontal shortening; e.g. Haschke et al., 2002; DeCelles et al., 2009; DeCelles and Graham, 2015; Balgord et al., 2021).

The model proposed in this work is supported by showing how the eastward migration of the deformation front coincides with aspects as increased accumulation rates in the retroarc foreland basins, temporal trends in magmatic activity, and compositional changes in the arc magmas based on the Hf isotopic composition. It is regarding this last issue that I consider the model has a major flaw, as the data is being carelessly interpreted. It must be noted that the Hf data presented in this work is from two sources: (1) the work of Jones et al. (2015), analyses performed in zircons from Cenozoic igneous rocks, and (2) the work of Capaldi et al. (2021), analyses performed in detrital zircons.

As described in Jones et al. (2015), Cenozoic igneous rocks in this region display a clear pattern of more enriched signatures (more “crustal like”) towards the east, as shown by mostly coeval units emplaced in the Frontal Cordillera and the Precordillera. This was ascribed by the authors to the presence of an old basement in the eastern regions (Grenvillian Basement – Cuyania Terrane?) responsible to imprint the enriched signature to the magmas that have transversed it in the eastern region (Precordillera). This had been also recognized earlier by Kay and Abbruzzi (1996) on this same region and by Muñoz et al. (2013) on central Chile (~34°S). A quick look at the data presented in the manuscript that considers the lithologies and the sample location, will reveal that: (1) rocks emplaced in the Frontal Cordillera describe, by themselves, a pattern towards enriched ƐHf signatures from ~28 to 6 Ma, and these data is the one mostly controlling the pattern in blue presented in the ƐHf vs Age diagram in the manuscript, (2) rocks emplaced in the Precordillera also define a pattern towards more enriched ƐHf signatures in time but in a comparatively lower ƐHf trend (more enriched than that of the Frontal Cordillera; note there are also minor juvenile inputs), and these are the samples that mostly control the pattern in red presented in the ƐHf vs Age diagram in the manuscript, (3) the detrital zircon data is a “disperse cloud” that encompasses both patterns previously described. The detrital data, for which there is no precise knowledge of the zircon source (location in the Andean orogen), is not reliable for the analysis/interpretations presented by the authors, as the “location” of the arc at any stage cannot be stablished. The ƐHf vs Age diagram presented by the authors is showing: (1) the basement control over the composition of Cenozoic Andean arc magmas, with those emplaced in the Frontal Cordillera being less enriched than those emplaced further east in the Precordillera (respectively the patterns in blue and in red in the diagram), and (2) the progressive enrichment that Cenozoic Andean magmas have suffered throughout the progressive crustal thickening that accompanies the construction of the modern orogen (the general patterns towards higher ƐHf signatures in time, in both areas).

Even though the models just described from Kay et al. (2005), Muñoz et al. (2013) and Jones et al. (2015) were not valid, the ƐHf vs Age interpretation of the manuscript is still not sustained. For example, the latest “arc event” recorded in ƐHf basis (~5-8 Ma; ƐHf: ~0 to -3) is indeed more juvenile than the immediately preceding event (~8-11 Ma; ƐHf: ~ -3 to -5), yet the samples defining the former are in a western location (Frontal Cordillera) respect the those defining the latter (Precordillera). This is the opposite to that indicated by the model of the manuscript, where the juvenile newly stablished arc is eastwards, and the same happens when looking in detail the older age data.

Regarding figures: (1) Fig. 1b is too overloaded with geological units and could be simplified, (2) in Fig. 2B the text within the diagrams is too small, (3) the third panel to the right in Fig 2 seems to be missing the “C”, (3) in Fig. 4 there should be an indication of what means/signals the black segmented line oval. I note also some problems with the supplementary material. First, the citation to the tables in the manuscript, in the Methods section, is wrong with respect to the labeling in the excel sheet containing the data. In the excel sheet itself: (1) “Table 1-Ign Ages” has text in Spanish and in English, (2) “Table 4 Zircon Hf” has no information on sample location. In this last case, such information can be readily found for the samples of Jones et al. (2015) in that work, but this is not the case for several samples from Capaldi et al. (2021; not found in that paper nor in the www.geochron.org; samples TAL06, TAL06m, TAL16, TAL14, TAL09, RAG01, RSJc01, ST02). This issue hampers a proper evaluation of the data analyzed in the manuscript and consequently of the conclusions drawn from it.

Best regards.

References

Balgord, E.A., Yonkee, W.A., Wells, M.L., Gentry, A. and Laskowski, A.K., 2021. Arc tempos, tectonic styles, and sedimentation patterns during evolution of the North American Cordillera: Constraints from the retroarc detrital zircon archive. Earth-Science Reviews, 216, p.103557.
Capaldi, T.N., McKenzie, N.R., Horton, B.K., Mackaman-Lofland, C., Colleps, C.L. and Stockli, D.F., 2021. Detrital zircon record of Phanerozoic magmatism in the southern Central Andes. Geosphere, 17(3), p.876-897.
DeCelles, P.G. and Graham, S.A., 2015. Cyclical processes in the North American Cordilleran orogenic system. Geology, 43(6), p.499-502.
DeCelles, P.G., Ducea, M.N., Kapp, P. and Zandt, G., 2009. Cyclicity in Cordilleran orogenic systems. Nature Geoscience, 2(4), p.251-257.
Haschke, M., Siebel, W., Günther, A. and Scheuber, E., 2002. Repeated crustal thickening and recycling during the Andean orogeny in north Chile (21–26 S). Journal of Geophysical Research: Solid Earth, 107(B1), p.ECV-6.
Jones, R.E., Kirstein, L.A., Kasemann, S.A., Dhuime, B., Elliott, T., Litvak, V.D., Alonso, R., Hinton, R. and Facility, E.I.M., 2015. Geodynamic controls on the contamination of Cenozoic arc magmas in the southern Central Andes: Insights from the O and Hf isotopic composition of zircon. Geochimica et Cosmochimica Acta, 164, p.386-402.
Kay, S.M. and Abbruzzi, J.M. Magmatic evidence for Neogene lithospheric evolution of the central Andean “flat-slab” between 30°S and 32°S. Tectonophysics, 259, 15-28.
Muñoz, M., Farías, M., Charrier, R., Fanning, C.M., Polvé, M. and Deckart, K. Isotopic shifts in the Cenozoic Andean arc of central Chile: Records of an evolving basement throughout cordilleran arc mountain building. Geology, 41(8), 931-934.

Version 1:

Reviewer comments:

Reviewer #1

(Remarks to the Author)
The authors have thoroughly addressed my previous comments and inquiries with detailed and thoughtful responses. I believe this manuscript will make an important contribution to the community studying orogenic systems, particularly

regarding the influence of high magmatic flux events on the location and magnitude of crustal deformation. Moreover, it offers a plausible explanation for the short-term episodes of high magmatic flux and crustal shortening observed in these systems. In my opinion, the manuscript is now ready for publication in its current form.

With regards,
Laura

Reviewer #2

(Remarks to the Author)
Please find my review in the attached pdf file.

Version 2:

Reviewer comments:

Reviewer #3

(Remarks to the Author)
Nature Communication, 572450
"Inboard advance of arc magmatism regulates mountain building in the Andes"

Reviewer: Devon A. Orme, Montana State University

Summary:

I read this paper and commented prior to reading the rebuttal to a previous review. In my first reading, I was very curious about the location of the data used in Figures 2 and 3 since they are not on Figure 1. This is important for readers to critically read the paper; the latitude and longitude of samples is reported in the supplementary material and figure 2 does include spatial information with the use of symbols. However, I recommend adding the sample locations used in Figure 3B to Figure 1. Below I expand on why this is necessary, especially in light of the previous reviews.
The zircon Hf and whole rock Nd trends are defined by the bedrock data. The detrital zircon Hf data broadly agree with the range of source regions, but take away from the well-defined bedrock pull-ups and pull-downs. In my opinion, the inclusion of the detrital data is fine for the overall interpretations, but is not as robust as the bedrock data. I comment below on the terminology around the detrital dataset at specific lines to help the authors rephrase some of their language around this.
The location of this data is central to the interpretations and differences between a reviewer and the authors. Given the implications of the proposed model to other convergent margins, linking Figure 1 to Figure 3 is essential for interpreting spatial trends. I had assumed the age of the circles and diamonds from Figure 3 (x-axis) correspond with the location of the data (Main Cordillera, Frontal, Pre, Sierras Pampeanas) as shown by the grey bars. However, the figure provided by Reviewer 2 clearly shows that some of the young (e.g., <10 Ma) zircons are from the Frontal Cordillera, not the regions farther east. For example, the Frontal Cordillera bedrock zircon Hf data at ~7 Ma range from ~ +1 to -3.5, and define the return to less evolved values (a "pull up" towards zero) on Figure 3B, but those data are outlined by the grey bar labeled Precordillera to the transition to the Sierras Pampeanas. At the same time, the whole rock Nd data from the Sierras Pampeanas support the pull up between ~8-3 Ma. This means that at <8 Ma within both the Frontal Cordillera and Sierras Pampeanas magmatism was moving towards 0, away from the evolved values recorded at ~ 10-8 Ma in the Precordillera. The mix of frontal cordillera and Sierras Pampeanas data with similar geochemical signatures alone does not support the conclusion that there is a progressively the spatial eastward trend, but does 1) support the model of progressively more evolved magmas through time and 2) when combined with the thermochronologic data, can be used to support spatial trends.
In summary, the authors present a provocative model for arc magmatism along convergent margins that will promote future interrogation of these margins and the model itself. This is a contribution that merits publication. However, I highly recommend the authors make use of variable symbols/colors for their bedrock and detrital samples so that the readers know the spatial distribution. This is done for the AFT and AHe data on Figure 2 well, but these locations are not shown on Figure 1. For the AFT and AHe data, a supplementary location map would suffice. However, given the emphasis on the Hf and Nd data in Figure 3, adding these data to Figure 1 and symbolizing the four geographic regions with different colors would result in a more transparent, and ultimately, more useful visual of the data to support the authors interpretations.

The authors are free to email me if any of my suggestions are unclear.

Best Wishes,
Devon

Specific Comments:

Line 44-45: Consider re-phrasing to be more direct. E.g., We hypothesize that short-term orogenic phases are linked to shifts in thermal…

Line 115: "consistent" is strong here considering the many outliers from the detrital record (Figure 3B). I recommend simply removing "consistent". There is a broad geochemical match.

If one removes the DZ EHf record, the bedrock record nicely shows the pull downs and pull-ups, albeit a bit more tightly constrained. Therefore, the emphasis should be placed on the bedrock record, with the detrital supporting interpretations, not broadening.

Line 166: Thermochronometric dates.

Lines 177-179 speak to shortening in the latest Oligocene-early Miocene, yet the curves in Panel C do not start until 20 Ma and there is no information on orthogonal subduction in panels B or C. Therefore, reference to this figure here could be clarified or removed. More importantly, panel B nicely shows the rapid increase in convergence rate at 24-23 Ma, which corresponds to the inboard shift in magmatism. This is crucial and not discussed in the text.

AHe reported data: The main text and figure caption must state these are mean dates from detrital and igneous bedrock datasets. This is crucial because it is a simplification of the data. Most samples have enormous ranges of single grains (e.g., AN01 has between 41-68 Ma, but a mean of 61…continuing down Supplementary File S4, many thermochronologists would argue these data should only be reported as single grain ages. I suspect if these are plotted, some of the trends dissipate. However, I understand the authors aim to share a ton of data clearly on Figure 2. Therefore, I recommend adding a supplementary figure to S4 that shows all the single age grains to eliminate any criticism that use of mean AHe is misleading. For quite a few AHe samples, the mean age is meaningless given the range of dates and control on those ages from geologic and mineral specific sources of error.

Figures:

Figure 1: The “arc front” polygon is a bit confusing when reading along with the text, since the data extent eastward of this. For example at 25-23 Ma, there is magmatism east of the “arc front”. Therefore, what does this line/polygon represent? It seems to be an oversimplication that is not needed. If used, it should mimic the text description of this plot over Lines 122-136.

Figure 1: At first, I thought the data came from the labeled basins, but the data extend into the main and coastal Cordillera. Once I got to line 184, it became the basin subsidence records of these areas are why they are labeled on Figure 1. Therefore, I am left wondering where the data are spatially in the study area? I highly recommend adding symbols on figure 1 that show where all the data from figure 2 are spatially located. Alternatively, this could be a supplemental figure.

Figure 2: The caption needs to state these are single mean AHe ages and central (?) AFT ages. Note: I assume these are central AFT and not pooled ages, but both the main text and supplementary table S5 need to make this clear.

Figure 3: Caption for c, please add “detrital” before zircon U-Pb ages. The label on panel C is quite small and this was unclear

Figure 4: This is well-crafted figure! However, it was unclear from the figure alone or caption that this is a proposed sequence until reading the discussion. To make this figure stand-alone (i.e. the citable figure that others may use in presentations, etc.), I recommend adding some sort of text (e.g., t1, t2, t3, and t4) or an arrow linking them (younger ----- > older).

Version 3:

Reviewer comments:

Reviewer #3

(Remarks to the Author)
Summary: The revised version of the manuscript addresses the concerns I raised with visualization of sample locations, use of mean thermochronologic dates, and wording around the detrital dataset. The authors have completed five tasks to address these:

1) Added all sample locations to Figure 1.
2) Modified symbols on Figure 3B to match Figure 1.
3) Added “Step 1”, “Step 2”, etc. to Figure 4, which makes it clear the hypothesized model through time.
4) The additional supplementary figures are very useful. Notably the addition of Supplementary Figure 3, which plots all single thermochronologic dates with their mean dates, justifies the use of mean dates for interpretation in the main text.
5) Minor word changes throughout the text, which are documented in the response to reviewer document.

I recommend this manuscript be accepted in its current form for publication. This paper will be a significant contribution to our scientific community studying orogenic systems.

Best Wishes,
Devon Orme

Open Access This Peer Review File is licensed under a Creative Commons Attribution 4.0 International License, which permits use, sharing, adaptation, distribution and reproduction in any medium or format, as long as you give appropriate credit to the original author(s) and the source, provide a link to the Creative Commons license, and indicate if changes were made.
In cases where reviewers are anonymous, credit should be given to 'Anonymous Referee' and the source.
The images or other third party material in this Peer Review File are included in the article’s Creative Commons license, unless indicated otherwise in a credit line to the material. If material is not included in the article’s Creative Commons license and your intended use is not permitted by statutory regulation or exceeds the permitted use, you will need to obtain permission directly from the copyright holder.
To view a copy of this license, visit https://creativecommons.org/licenses/by/4.0/

Response to reviewer comments/suggestions

This document provides a detailed, point-by-point response to comments from reviewers associated with the original submission of this manuscript to Nature Communications (NCOMMS-24-86680).
Our responses below are shown in **BOLD** and line numbers refer to the revised manuscript.

REVIEWER COMMENTS

Reviewer #1 (Remarks to the Author):

This contribution by Capaldi and coauthors integrates geo/thermochronological, geochemical and stratigraphic records of Cenozoic arc magmatism to shed light on the interplay between subduction dynamics, arc magmatism, back-arc crustal shortening and flexural subsidence in Andean-type subduction orogens. To achieve this, a compilation of igneous rock crystallization ages, combined with zircon U-Pb geochronology and εHf isotopic compositions, provides valuable insights into the mechanisms governing these complex geological processes.

The manuscript presents a compelling case for the role of short-term (5-8 Myr) tectonic-magma cycles in controlling deformation and foreland basin subsidence. This represents a significant advancement over previous models that have primarily focused on longer (10-20 Myr) episodes of magmatism, deformation and crustal thickening. The discussion on how these shorter cycles particularly affect regions with lower magnitudes of shortening is insightful and adds an important dimension to our understanding of Andean orogenesis.

Overall, this paper is well-framed, clear, concisely written and technically sound. However, I have a few suggestions that might enhance its clarity and impact:
We thank the reviewer for their insights and critical evaluation of our work.

The timing of εHf isotopic variations could be more precisely constrained. For instance, in Figure 3, while the trend between 28 Ma and 22 Ma is well-explained, the period between 25-22 Ma shows both positive and negative εHf values, suggesting contributions from both depleted-mantle and enriched-crustal melts. A discussion of the possible causes in the text for this mixed signal would be beneficial.
You make a good point. We have elaborated on the potential for magma mixing

during progressive change in magmatic sources during this 1 to 2 Myr overlap in zircon εHf values. See lines 135-138.

The second pull-down phase (19-13 Ma) is linked to the expansion of arc magmatism into the Precordillera. However, the isotopic pull-down appears to begin after 16-17 Ma. A clarification of this apparent discrepancy would be helpful.
Yes there are apparent lag times between arc advance, retroarc shortening, and resulting change in arc magmatism. Our multi-phase model attempts to address the sequence of events where arc migration is temporally followed by shortening which induces a high-flux event in the magmatic arc. Although we don't directly link the Central Andes data to the model the 16 Ma isotopic pull down follows arc expansion around 19 Ma, we address the temporal overlap in both depleted and enriched values reflecting magma mixing during changes in magmatic sources. Line 130-140.

The subsidence of the Talacasto Basin, located far from the thrust front in the Main Cordillera, is intriguing. The absence of similar subsidence evidence in the Calingasta and Manantiales Basins at the eastern foot of the Frontal Cordillera also warrants further discussion.
The seemingly early initiation and relatively higher subsidence rates of the Talacasto Basin are the result of the Oligocene-Miocene erg system that developed in the Precordilleran region (Starck et al., 2023). When looking along strike the Talacasto basin history is comparable to the Las Juntas (La Troya) system to the North (Reynolds et al., 1990; Cicioli et al., 2014; Mackaman-Lofland et al., 2020). But you bring up a good point about Manantiales and Calingasta, why don't we see a similar subsidence pattern? One option could be our age constraints of the lower intervals are relatively sparse. In Mananitales the basal Rio de Los Patos Formation has good age constraints in the lower part dating back to the Eocene but lack constraints in the upper unit (Suriano et al., 2023). The same can be said about the Calingasta location where there several > 10 Myr gaps in age data in the lower section, which provides low resolution of foreland basin initiation there (Capaldi et al., 2020). In this context, we may be "missing" the data that would indicate basin initiation in line with what is known from Talacasto. Another option, albeit more speculative, may involve as-yet-underappreciated complex paleotopography underlying the basin system. To the east, in the Bermejo Basin, we observed along strike variation in the timing of basin initiation as the result of paleotopographic highs (Plonka et al., 2023). Perhaps Talacasto preserves an appreciable amount of low paleotopography that was infilled by eolian facies, while the western Calingasta and Manantiales Basins

represented a net-transport system until flexurally-induced subsidence initiated in the early Miocene. All of this is intended to reinforce the point that Talacasto fits into the paradigm of our understanding of the foreland basin system here, but we have considered these nuances and agree with the reviewer regarding their importance. However, given the target audience and primary focus for this manuscript, we will hold off on such detailed speculation until the sequel manuscript.

Capaldi, T.N., Horton, B.K., McKenzie, N.R., Mackaman-Lofland, C., Stockli, D.F., Ortiz, G. and Alvarado, P., 2020. Neogene retroarc foreland basin evolution, sediment provenance, and magmatism in response to flat slab subduction, western Argentina. *Tectonics*, *39*(7), p.e2019TC005958.

Ciccioli, P.L., Marenssi, S.A., Limarino, C.O., 2014b. Petrology and provenance of the Toro Negro formation (Neogene) of the Vinchina broken-foreland basin (central Andes of Argentina). J. S. Am. Earth Sci. 49, 15–38.

Mackaman-Lofland, C., Horton, B.K., Fuentes, F., Constenius, K.N., Ketcham, R.A., Capaldi, T.N., Stockli, D.F., Ammirati, J.B., Alvarado, P., Orozco, P., 2020. Andean mountain building and foreland basin evolution during thin- and thick-skinned Neogene deformation (32□ 33◦S). Tectonics 39. https://doi.org/10.1029/2019TC005838.

Plonka, Z.C., Capaldi, T.N., Odlum, M.L., Mackaman-Lofland, C., Ortiz, G. and Alvarado, P., 2023. Along-strike tectonic evolution of the Neogene Bermejo foreland basin and Eastern Precordillera thrust front, Argentina (30-32° S). *Journal of South American Earth Sciences*, *129*, p.104521.

Reynolds, J.H., Jordan, T.E., Johnson, N.M., Damanti, J.F., Tabbutt, K.D., 1990. Neogene deformation of the flat-subduction segment of the Argentine-Chilean Andes: Magnetostratigraphic constraints from Las Juntas, La Rioja province, Argentina. Geol. Soc. Am. Bull. 102, 1607–1622.

Starck, D., Capaldi, T.N., Fuentes, F. and Horton, B.K., 2024. Tectonic and climatic significance of Oligocene-Miocene eolian sandstones in the Andean foreland basin of Argentina. *Journal of South American Earth Sciences*, *134*, p.104758.
Figure 4: This is an excellent and self-explanatory figure. I suggest incorporating a time-referencing framework from the Southern Central Andes example to provide additional and specific context for different phases.

Suriano, J., Lossada, A.C., Mahoney, J.B., Tedesco, A.M., Limarino, C.O., Giambiagi, L.B., Mazzitelli, M.A., Mescua, J.F., Lothari, L., Quiroga, R., 2023. The southern extension of the Eocene Andean orogeny: New sedimentary record of

the foreland basin in the southern Central Andes at 32◦S. Basin Res. https://doi.org/10.1111/bre.12803.

Figure 4: This is an excellent and self-explanatory figure. I suggest incorporating a time-referencing framework from the Southern Central Andes example to provide additional and specific context for different phases.

Thank you, and you make a good point. Previous iterations of the model were based on direct constraints from the Southern Central Andes. However, to allow the model to be more transportable to other systems we decided to create a more generic, prediction-based model that leverages the datasets from the Southern Central Andes but does not explicitly call upon specific temporal events, structures (i.e. Frontal Cordillera), or basin systems (i.e. Bermejo).

Additional points for consideration on this Figure:

Why is there no lithospheric mantle beneath the arc axis in Step 1? I suggest changing the figure or explaining why not?
We have added a thin lithospheric mantle in the forearc regions in figure 4A and 4B.

In Step 2, could you elaborate on how fluids migrate to the retroarc region?
We elaborated on this point in Step 1 Lines 223-224.

In Step 3, does the model require a normal or thin crust? Could you expand on how flexural subsidence in the foreland relates to shortening?
That's a great question, given that most Cordilleran systems have experienced a prolong phase of crustal extension that precedes orogenesis we suspect normal or thin crust is typically a common starting condition. For example, North American Cordillera records Triassic to Jurassic extensional to neutral stress regime followed by Sevier shortening. The Andes also reflect significant Triassic-Jurassic to Cretaceous crustal thinning, obviously with some degree of along-strike variability.

Minor issues:
Abstract: Clarify why magmatism migrates in your model and whether slab flattening is a necessary condition or not.
Due to the Journal's restricted word count for the abstract section we don't have

the space to fully address your comment in the abstract. However, we have added new discussion text in the "Implications for Andean-Type (Cordilleran) Orogenic Systems" that fleshes out the role of flat-slab and arc migration. See lines 293-306.

Line 51: Further explain why landward arc magmatism migration enhances fluid flux.

We have rephrased this sentence to "Such landward migration expands slab dewatering further into the mantle wedge that enhances fluid flux and hydration of overriding foreland lithosphere…".

Lines 67-69: The phrase "spatial and temporal variations" could be more specific.
We have rephrased the sentence to "Because the aforementioned interactions vary over a range of spatial and temporal scales,…" .

Line 104: The study focuses on the relationship between arc magmatism and retroarc crustal shortening, rather than broad crustal deformation. Please correct.
We changed the text from "deformation" to "crustal shortening".

Lines 115-117: Address the proposed crustal extension during the late Oligocene-Early Miocene (25-20 Ma).
You bring up a good point. There is still a great debate on timing and magnitude of extension preceding Andean contraction. However, in this section we try and focus the text on observation of the arc position and composition through time avoiding interpreting the tectonic regime. We link structural activity in the next section "Retroarc Shortening and Flexural Response" where we highlight the transtensional regime in the Oligocene.

Lines 119-129: Discuss the isotopic pull-up periods (21-18 Ma, 13-11 Ma, 7-6 Ma), which are visible in Figure 3 but not explained in the text.
We have added a summary paragraph to this section highlighting key trends in zircon geochemistry and elaborate on potential drivers. See lines 130-140.

Lines 157-158: Clarify the timing of major subsidence and its correlation with the Main Cordillera shortening.
We have rephrased this sentence to emphasize which basin sites are recording subsidence and refined timing of foreland basin initiation. See Lines 168-173.

Line 160: Define "high-angle reverse faults" more clearly.
We removed “high-angle” for clarity.

Lines 204-206: Could you specify which retroarc regions are weakened by slab-derived fluids?
We have rephrased the sentence to indicate weakening occurs proximal to melt and fluid input.

Line 210: The term "High-Flux Events (HFEs)" should be introduced earlier with an explanation.
We now introduce the concept of high-flux-events in the “Magmatic Arc Activity and Composition” section. See lines 110-114.

Figure 1: Add basin names to the caption.
Agreed, we have added the basin localities within the map explanation.

Overall, this is a valuable contribution to the field. However, addressing the points above would further strengthen the manuscript and clarify key interpretations. I would strongly encourage publication, following these refinements.

Best regards,
Laura Giambiagi

Reviewer #2 (Remarks to the Author):

In this letter I send you my comments on the paper entitled "Inboard advance of arc magmatism regulates mountain building in the Andes" coauthored by Capaldi, T., Horton, B., Mackaman-Lofland, C., Fuentes, F. and Ortiz, G.

This work explores the interactions and feedbacks among subduction, magmatism, and crustal deformation in a continental margin setting taking as a case study the southern Central Andes, mostly between ~29°-33°S. It is based on an extensive compilation of data that includes igneous ages, apatite (U-Th-Sm)/He and fission track thermochronology, and zircon Hf isotopic composition, in addition to calculations of sediment accumulation rates and deformation front reconstructions also based on the information reported by previous studies. The main text of the manuscript is well organized, the 4 figures included are appropriate to illustrate the issues discussed, and there is an extensive supplementary material which provides the data on which the work is based. I do consider that the figures and the supplementary material could be improved (see below).

As a main result, the work proposes a cyclic model that links short-term orogenic phases with shifts in thermal and rheological processes with the following sequential events: (1) arc magmatism weakens the crust, (2) the latter can then be deformed mostly by retroarc shortening with westward underthrusting, (3) crustal thickening prompts slab shallowing and a consequent eastward arc migration, (4) the newly allocated arc starts the cycle again. The model is very appealing as it offers a linked explanation of major processes observed throughout Andean orogenic construction: the eastward migration of arc magmatism and of deformation. In addition, as stated in the manuscript itself, the model focuses on internal orogenic cycles much shorter (<10 Myr) and of less magnitude (<50-150 km horizontal shortening) than those already covered in previous works (>20-50 Ma and >200-300 km horizontal shortening; e.g. Haschke et al., 2002; DeCelles et al., 2009; DeCelles and Graham, 2015; Balgord et al., 2021).

The model proposed in this work is supported by showing how the eastward migration of the deformation front coincides with aspects as increased accumulation rates in the retroarc foreland basins, temporal trends in magmatic activity, and compositional changes in the arc magmas based on the Hf isotopic composition. It is regarding this last issue that I consider the model has a major flaw, as the data is being carelessly interpreted. It must be noted that the Hf data presented in this work is from two sources: (1) the work of Jones et al. (2015), analyses performed in zircons from Cenozoic igneous rocks, and (2) the work of Capaldi et al. (2021), analyses performed in detrital zircons.

We thank the reviewer for their thorough comments regarding the data handling and presentation. We have revised each figure and refined the supplemental data material. We address each comment individually below.

As described in Jones et al. (2015), Cenozoic igneous rocks in this region display a clear pattern of more enriched signatures (more “crustal like”) towards the east, as shown by mostly coeval units emplaced in the Frontal Cordillera and the Precordillera. This was ascribed by the authors to the presence of an old basement in the eastern regions (Grenvillian Basement – Cuyania Terrane?) responsible to imprint the enriched signature to the magmas that have transversed it in the eastern region (Precordillera). This had been also recognized earlier by Kay and Abbruzzi (1996) on this same region and by Muñoz et al. (2013) on central Chile (~34°S). A quick look at the data presented in the manuscript that considers the lithologies and the sample location, will reveal that: (1) rocks emplaced in the Frontal Cordillera describe, by themselves, a pattern towards enriched ƐHf signatures from ~28 to 6 Ma, and these data is the one mostly controlling the pattern in blue presented in the ƐHf vs Age diagram in the manuscript, (2) rocks emplaced in the Precordillera also define a pattern towards more enriched ƐHf signatures in time but in a comparatively lower ƐHf trend (more enriched than that of the Frontal Cordillera; note there are also minor juvenile inputs), and these are the samples that mostly control the pattern in red presented in the ƐHf vs Age diagram in the manuscript,

Yes, you make excellent points here. We have observed a similar pattern in longitude vs. zircon ƐHf for a Phanerozoic dataset in the Southern Central Andes (Capaldi et al., 2021- Fig. 10). We determined that inboard arc migration over 800 km produces a shift towards more enriched composition as previously observed by (Chapman et al., 2017; Chapman and Ducea, 2019). The Jones et al., 2015 dataset is spatially limited to -70°S to -69°S: samples were collected mainly in the Frontal Cordillera with one sample (RJ11A5) being truly in the Precordillera across the speculated Chilenia-Cuyania terrane boundary. The variability of average zircon ƐHf in the Frontal Cordillera over the past 300 Myr ranges between +4 and -4 in ƐHf space (Capaldi et al., 2021- Fig. 10). Your point that the zircon ƐHf is purely recording a spatial trend is true to a certain extent, however this mechanism alone cannot fully explain the temporal variations observed in the data. The Jones et al., 2015 samples in the Frontal Cordillera exhibit isotopic pull-downs (samples PC14, RJ11A10, RJ11A11, RJ11A14 between 24-21 Ma; samples RJ11A17, RJ11A15, DI095, MQ33 between 10-6 Ma) and pull-ups (samples SP80, 1026, RF62 between 19-17 Ma; and sample RJ11A7 at 12 Ma). We interpret the temporal variation in the zircon ƐHf to record changes in the magmatic sources driven by crustal thickening associated with underthrusting of fertile crustal material during phases of shortening. We acknowledge your point of a protracted

30 Myr trend in zircon ƐHf signifying enriched magmatic sources that record progressive crustal thickening during Andean Mountain building and spatial trends of the arc across different magmatic sources, we have expanded the text in lines 130-140.

Capaldi, T.N., McKenzie, N.R., Horton, B.K., Mackaman-Lofland, C., Colleps, C.L. and Stockli, D.F., 2021. Detrital zircon record of Phanerozoic magmatism in the southern Central Andes. Geosphere, 17(3), pp.876-897.

Chapman, J.B., Ducea, M.N., Kapp, P., Gehrels, G.E. and DeCelles, P.G., 2017. Spatial and temporal radiogenic isotopic trends of magmatism in Cordilleran orogens. *Gondwana Research*, *48*, pp.189-204.

Chapman, J.B. and Ducea, M.N., 2019. The role of arc migration in Cordilleran orogenic cyclicity. *Geology*, *47*(7), pp.627-631.

(3) the detrital zircon data is a "disperse cloud" that encompasses both patterns previously described. The detrital data, for which there is no precise knowledge of the zircon source (location in the Andean orogen), is not reliable for the analysis/interpretations presented by the authors, as the "location" of the arc at any stage cannot be stablished. The ƐHf vs Age diagram presented by the authors is showing: (1) the basement control over the composition of Cenozoic Andean arc magmas, with those emplaced in the Frontal Cordillera being less enriched than those emplaced further east in the Precordillera (respectively the patterns in blue and in red in the diagram), and (2) the progressive enrichment that Cenozoic Andean magmas have suffered throughout the progressive crustal thickening that accompanies the construction of the modern orogen (the general patterns towards higher ƐHf signatures in time, in both areas).

We acknowledge the hesitancy to interpret arc processes using the sedimentary record. However, the detrital zircon geochronology coupled with geochemistry has been shown to be a robust tool in tracking the timing and pattern of magmatism both in continental and oceanic igneous systems (e.g., Portner et al., 2011; Barth et al., 2013; Surpless et al., 2019; Balgord et al., 2021; Schwartz et al., 2021). The detrital record may lack direct correlation to a specific eruption center, but these data (1) fill in the gaps left by the "petrologist effect" in bedrock sampling non-altered igneous units, (2) efficiently sample large areas of catchment bedrock sources, and (3) preserve the magmatic record from rock units that have been completely removed from the landscape during mountain building and denudation in arc regions. We view the combination of both bedrock and detrital zircon U-Pb geochronology and ƐHf geochemistry as a strength, not a weakness. Not only does the detrital data overlaps the bedrock

data in ƐHf space, but the data also importantly fills in the gap at ~15-10 Ma from the Jones et al., 2015 data. The sandstones (samples VILE10, VILE11, VN05, TAL06, TAL16, TAL09, AMP03, MGN04) are sampled within the context of measured stratigraphic sections that are characterized by volcanic arc sediment provenance indicators (Levina et al., 2014; Capaldi et al., 2020; Plonka et al., 2023), which supports the syndepositional contribution of arc material from the proximal Andean arc into the retroarc basins of Argentina.

Balgord, E.A., Yonkee, W.A., Wells, M.L., Gentry, A. and Laskowski, A.K., 2021. Arc tempos, tectonic styles, and sedimentation patterns during evolution of the North American Cordillera: Constraints from the retroarc detrital zircon archive. *Earth-Science Reviews*, *216*, p.103557.

Barth, A.P., Wooden, J.L., Jacobson, C.E. and Economos, R.C., 2013. Detrital zircon as a proxy for tracking the magmatic arc system: The California arc example. *Geology*, *41*(2), pp.223-226.

Capaldi, T.N., Horton, B.K., McKenzie, N.R., Mackaman-Lofland, C., Stockli, D.F., Ortiz, G. and Alvarado, P., 2020. Neogene retroarc foreland basin evolution, sediment provenance, and magmatism in response to flat slab subduction, western Argentina. *Tectonics*, *39*(7), p.e2019TC005958.

Levina, M., Horton, B.K., Fuentes, F. and Stockli, D.F., 2014. Cenozoic sedimentation and exhumation of the foreland basin system preserved in the Precordillera thrust belt (31–32 S), southern central Andes, Argentina. *Tectonics*, *33*(9), pp.1659-1680.

Plonka, Z.C., Capaldi, T.N., Odlum, M.L., Mackaman-Lofland, C., Ortiz, G. and Alvarado, P., 2023. Along-strike tectonic evolution of the Neogene Bermejo foreland basin and Eastern Precordillera thrust front, Argentina (30-32° S). *Journal of South American Earth Sciences*, *129*, p.104521.

Portner, R.A., Daczko, N.R., Murphy, M.J. and Pearson, N.J., 2011. Enriching mantle melts within a dying mid-ocean spreading ridge: Insights from Hf-isotope and trace element patterns in detrital oceanic zircon. *Lithos*, *126*(3-4), pp.355-368.

Surpless, K.D., Clemens-Knott, D., Barth, A.P. and Gevedon, M., 2019. A survey of Sierra Nevada magmatism using Great Valley detrital zircon trace-element geochemistry: View from the forearc. *Lithosphere*, *11*(5), pp.603-619.

Schwartz, T.M., Surpless, K.D., Colgan, J.P., Johnstone, S.A. and Holm-Denoma, C.S., 2021. Detrital zircon record of magmatism and sediment dispersal across the North American Cordilleran arc system (28–48° N). *Earth-Science Reviews*, *220*, p.103734.

Even though the models just described from Kay et al. (2005), Muñoz et al. (2013) and Jones et al. (2015) were not valid, the ƐHf vs Age interpretation of the manuscript is still not sustained. For example, the latest "arc event" recorded in ƐHf basis (~5-8 Ma; ƐHf: ~0 to -3) is indeed more juvenile than the immediately preceding event (~8-11 Ma; ƐHf: ~ -3 to -5), yet the samples defining the former are in a western location (Frontal Cordillera) respect the those defining the latter (Precordillera). This is the opposite to that indicated by the model of the manuscript, where the juvenile newly stablished arc is eastwards, and the same happens when looking in detail the older age data.

We have modified figure 4 to highlight arc widening process associated with arc front migration. Thereby magmatism is occurring in a broader region in the arc and not just at the arc front.

Regarding figures: (1) Fig. 1b is too overloaded with geological units and could be simplified,

You make a great point. We have reduced the number of geological units on the map and cross-section from eleven to seven. We hope the revised figure 1 is more comprehensible to readers.

(2) in Fig. 2B the text within the diagrams is too small, (3) the third panel to the right in Fig 2 seems to be missing the "C",

Good catch. We have revised figure 2 by increasing text size and adding the "C" label.

(3) in Fig. 4 there should be an indication of what means/signals the black segmented line oval.

We added a leader line between the dashed ovals and "magma source" label to clarify the symbols significance.

I note also some problems with the supplementary material. First, the citation to the tables in the manuscript, in the Methods section, is wrong with respect to the labeling in the excel sheet containing the data. In the excel sheet itself:

Good catch. We have modified the sequence of tables in the supplemental file and updated the referencing in the text.

(1) "Table 1-Ign Ages" has text in Spanish and in English,

We have translated most of the Spanish to English. Some Spanish words refer to their formal Formation, Group, Complex, or unit names which remain in Spanish.

(2) “Table 4 Zircon Hf” has no information on sample location. In this last case, such information can be readily found for the samples of Jones et al. (2015) in that work, but this is not the case for several samples from Capaldi et al. (2021; not found in that paper nor in the www.geochron.org; samples TAL06, TAL06m, TAL16, TAL14, TAL09, RAG01, RSJc01, ST02). This issue hampers a proper evaluation of the data analyzed in the manuscript and consequently of the conclusions drawn from it.

We have modified “Supplemental Table 2 Zircon Hf” to include sample location, sample Formation name, and zircon grain number. Additionally, we have removed samples that are not used in the data plots. We hope the additional metadata provides all the information needed to recreate our analysis.

Response to Reviewer #2

Provided below are the original reviewer comments followed by our detailed, point-by-point responses concerning the resubmitted manuscript to Nature Communications (NCOMMS-24-86680).

Reviewer 2 Comments

In this new version of the manuscript, the authors have taken care of all the minor comments I made in my previous review, yet our disagreement with the interpretation of the Hf isotopic data remains. I see two main issues in this discussion: (i) the scopes and limitations of data from detrital zircons, and (ii) the interpretation of the ƐHf(t) data in terms of the spatial temporal-compositional analysis. I refer to each of these in the following.

Regarding the detrital zircon data, in my previous review I questioned its use in the model presented by the authors as they have no control over the precise original location of such data in space in the Andean orogen. The authors have replied that it has been shown that this is still a robust tool in tracking the timing and pattern of magmatism. They also add that the data fill in the gaps left by the "petrologist effect" (in bedrock sampling non-altered igneous units) and that it efficiently samples large areas of catchment bedrock sources. I agree with all these statements, yet still the core of my initial critic remains: the detrital data cannot be used for spatial interpretations about the arc, regarding its location and composition at any given time, because there is no control on the original location of such data. I include below a graph of ƐHf(t) vs t diagram (Fig. A) using the data presented by the authors where the detritic data has been separated from the bedrock data in the symbology (small gray dots). As indicated by the authors, the detrital data indeed totally overlaps with the bedrock data and I agree that those zrcs probably come from the Precordillera and the Frontal Cordillera areas. The issue here is that there is no precise control of where those zrcs come from among and within the mentioned areas. The model presented by the authors is fully dependent on a spatial variable, in the location and composition of the arc magmas in time, so detrital data cannot be used for supporting inferences in that respect.

Regarding the interpretations of the ƐHf(t) data, the model presented by the authors
predicts a cycle that: (1) starts with arc magmas at a given location with high ƐHf (mantle-like), (2) then the arc magmas show a decreasing ƐHf (more crustal-like) at that same location, (3) then the arc migrates eastwards and magmas show a high ƐHf (mantle-like) starting the cycle again. This model is clearly depicted in Fig. 4 of the manuscript in the previous and the current version of this work. In my previous review I argued against this interpretation based on the actual location of the samples recording the arc location and composition in time (bedrock samples) along with considering previous works in the area.

I indicated that Cenozoic igneous rocks in this region show more enriched signatures (more "crustal like") towards the east, as shown by mostly coeval units emplaced in the Frontal Cordillera and the Precordillera. This was ascribed by Jones et al. (2015), and previously by Kay & Abbruzzi (1996), to the presence of an old basement in the eastern regions (Grenvillian Basement – Cuyania Terrane?) responsible to imprint the enriched signature to the magmas that have transversed it in the eastern region (Precordillera). In their reply, the authors recognize that in this region Andean magmas show progressively more enriched compositions towards the east, but they question my arguments that the ƐHf(t) trend is purely recording a spatial trend indicating that this mechanism alone cannot fully explain the temporal variations observed in the data. Regarding this, there are some misinterpretations on my comments: I do argument that there is a spatial control on the composition of arc magmas, they become generally more enriched towards the east, but this is in addition over imprinted by the general increase in crustal thickness throughout the Andes construction in Cenozoic (from ~22-18 Ma to the present; see Giambiagi & Ramos, 2002; Farías et al., 2010) which adds an increasing crustal signature to progressively younger magmas (lower ƐHf(t) signatures). The ƐHf(t) vs t diagram of Fig. A below shows this very clear for the Frontal Cordillera: igneous rocks emplaced there define a trend towards enriched signatures in time. Moreover, I have included below a map showing the location of all samples discussed in the Capaldi et al. study (detrital and bedrock; Fig. B), where it can be seen that the "trend" for the Frontal Cordillera is constrained by samples very restricted in space (westernmost samples of all those considered in this study; blue dots in Fig. A, B). Regarding rocks emplaced towards the east (in the Precordillera and Precordillera/Frontal Cordillera), as I mentioned in my previous review, they also define a trend towards more enriched ƐHf(t) signatures in time but at a comparatively lower ƐHf values (more enriched than those of the Frontal Cordillera) with minor juvenile inputs.

I must refer to two arguments the authors presented in their reply to discredit the interpretations I discussed above (Jones et al., 2015; Kay & Abbruzzi, 1996). First, they argue that the Jones et al. (2015) dataset is spatially limited to -70°S to -69°S: samples were collected mainly in the Frontal Cordillera with one sample (RJ11A5) being truly in the Precordillera across the speculated Chilenia-Cuyania terrane boundary. I'm not sure where the authors point with this argument: the Jones et al. (2015) data is the only data with spatial location control they have for their model, discrediting this dataset discredits their own spatial-temporal compositional interpretations. Moreover, the detrital data they present does not add much to the story, given its location and the arguments I presented before regarding such data (see Fig. A, B). Whether limited or not, the Jones et al. (2015) data shows clearly the trends I discussed in the previous paragraph and that are illustrated in Fig. A below. The second argument presented by the authors is that the variability of average zircon ƐHf in the Frontal Cordillera over the past 300 Myr ranges between +4 and -4 in ƐHf space (Capaldi et al., 2021- Fig. 10). I agree, the Fig A below

shows this clearly for the Cenozoic: magmas that have transversed the Frontal Cordillera area show a full range of ƐHf(t) values between -4 and +4. The issue here is that, within this range, during Cenozoic there is a temporal evolution to lower signatures (more crustal-like) in younger ages, which is most likely related to the increasing crustal thickness that accompanies the construction of the modern orogen.

In their reply, the authors also present their interpretation of the Jones et al. (2015) data in detail, indicating that samples in the Frontal Cordillera exhibit isotopic pull-downs (samples PC14, RJ11A10, RJ11A11, RJ11A14 between 24-21 Ma; samples RJ11A17, J11A15, DI095, MQ33 between 10-6 Ma) and pull-ups (samples SP80, 1026, RF62 between 19-17 Ma; and sample RJ11A7 at 12 Ma). The problem here is what they are considering as located in the Frontal Cordillera or elsewhere. Setting aside "names", Figs. A and B below show that within the full age range considered the spatially restricted group of westernmost samples (in blue in the figures) is comparatively more depleted (mantle-like) than coeval magmas emplaced in eastern regions except for local juvenile inputs (2 samples at ~24 and 12 Ma).

The authors have added to the new version of the manuscript a paragraph that describes general aspects of the arc magmas composition in the studied region. They indicate there that: "Coupled zircon geochemical and age data exhibit a progressive decrease in zircon εHf values over the last 30 Myr (Fig. 3B). When considered alongside with bedrock sample locations (Fig. 2A), a spatial relationship emerges: relatively asthenospheric (juvenile) compositions are preferentially located near the trench, in contrast while more lithospheric (evolved) contributions are concentrated toward the craton (Fig. 3B). Superimposed on this 30 Myr trend are higher-frequency phases (5-8 Myr) in which the relatively depleted positive εHf values represent background arc magmatism generated by juvenile mantle sources, which were perturbed during isotopic pull-downs phases reflecting greater contribution of evolved crustal sources (Fig. 3B). A 1 to 2 Myr temporal overlap in both depleted and enriched values are observed at the onset of the isotopic pull-down indicating magma mixing processes during changes in magmatic source (Hawkesworth and Kemp, 2006)". The processes described here, and the model depicted in Fig. 4 of the manuscript, are not supported by the data. For example, there is not a single sample from the spatially restricted westernmost group of the Fontal Cordillera that describes an "isotopic pull down" from the general trend they described through Cenozoic (blue spots in Fig A); the comparatively lower ƐHf signatures are described exclusively by samples located to the east. Moreover, the model presented by the authors predicts a zigzag ƐHf trend marked by high and low values developing sequentially in time towards the east: this is by no means described by data with spatial control (Fig A, B).

In summary, I consider that the model presented by the authors is attractive and can explain several geological observations. However, in my opinion the magmatic

evolution this model entails, in terms of the sequential variation in arc location and its composition, is not supported by the data.

Response to Reviewer 2

We appreciate Reviewer 2's evaluation of our manuscript. Their detailed comments have helped us clarify key points in our revised manuscript in support of our tectonic reconstruction of the southern central Andes, with broader implications for Cordilleran systems.

Reviewer 2 raises two main concerns regarding our interpretation of the isotopic data: (1) the spatial limitations of detrital zircon datasets, and (2) the validity of our multi-phase model for magmatic source evolution. They argue that detrital data, while useful for tracking temporal trends, cannot reliably constrain spatial relationships due to uncertain provenance. Additionally, they propose a spatially anchored model in which magmatic compositions become progressively more enriched toward the east, overprinted by a gradual increase in crustal thickness through time, which results in a smooth, monotonic decline in ƐHf values. In contrast, our model emphasizes episodic shifts in magmatic source linked to deformation-driven processes such as underthrusting and arc widening. Below, we carefully consider the alternative conceptual model presented by Reviewer 2, point out several limitations in their model, and show how our interpretation (utilizing results from both bedrock and detrital materials) provides a more compelling case that is supported by extensive isotopic, geochronologic, and structural/stratigraphic data.

1. Spatial Control and the Role of Detrital Zircon Geochemistry:

While we acknowledge that detrital datasets can be spatially diffuse and subject to mixing, this limitation does not preclude their utility—particularly when interpreted in tandem with spatially constrained in-situ bedrock data. In fact, the close correspondence between detrital zircon compositions and those of Frontal Cordillera and Precordillera igneous centers reinforces their provenance and validates their use in tracking temporal shifts in magmatic geochemistry.

Rather than dismissing detrital data outright, we argue that its strength lies in capturing basin-wide signals of magmatic evolution, especially in regions where bedrock exposures are discontinuous, structurally complex, or biased by sampling limitations. When integrated with latitudinally and longitudinally resolved bedrock datasets, detrital zircon geochemistry provides a robust framework for reconstructing temporal and geochemical trends across the arc system. This integrated approach enables us to detect episodic shifts in magmatic source and arc position that would be obscured by relying solely on bedrock samples.

We have therefore retained the detrital dataset in our revised analysis, not as a spatial proxy in isolation, but as an important line of evidence that enhances the resolution of magmatic trends when combined with in-situ whole-rock and bedrock zircon isotopic data.

2. Temporal Variability in Magmatic Sources:

Reviewer 2 proposes an alternative conceptual model that makes two key predictions. First, a prolonged, linear decrease in zircon ƐHf values in the Frontal Cordillera—such that successive magmatic phases yield progressively lower values through time. Second, in contrast, Precordillera magmatism is predicted to follow a parallel trend, offset towards more negative values, reflecting its recycled, evolved crustal signature.

However, this linear trend is not supported by the data. Instead, both Frontal Cordillera and Precordillera datasets exhibit a step-wise pattern: juvenile (positive) εHf values shift toward more enriched (negative) compositions, then rebound to more juvenile values. To reinforce this interpretation, we have incorporated whole-rock ƐNd data from the Frontal Cordillera, Precordillera, and Sierras Pampeanas, complied by Kay et al., (1999) and drawn from her earlier studies (Kay et al., 1991; Kay and Gordillo, 1994; Kay and Abbruzzi, 1996).

Given the well-established coherence between Hf-Lu and Nd-Sm isotopic systems (e.g., Patchett et al., 1981; Vervoort et al., 2000; Chapman et al., 2017), these εNd data provide an independent and spatially constrained line of evidence for variability in magmatic geochemistry. The combined zircon εHf and whole-rock εNd datasets clearly demonstrate step-wise isotopic pull-downs and subsequent pull-ups in the magmatic systems. These short-timescale fluctuations are superimposed on the broader 30 Myr trend toward more enriched signatures, supporting our interpretation of episodic crustal thickening and source evolution during discrete shortening phases (see Fig. A).

3. Misunderstanding of the Multi-Phase Model

We respectfully disagree with Reviewer 2's interpretation of our multi-phase model. Reviewer 2 summarizes our model as predicting a cycle that *" (1) starts with arc magmas at a given location with high ƐHf (mantle-like), (2) then the arc magmas show a decreasing ƐHf (more crustal-like) at that same location."* This characterization misrepresents the spatial dynamic central to our model.

Our model emphasizes the evolving relationship between the arc front and the deformation front, highlighting the lag between initial magmatic migration inboard and the onset of deformation. In this framework, slab-derived fluids weaken the foreland lithosphere, inducing deformation that generates upper crustal loading and flexural subsidence. This is coeval with middle to lower crustal underthrusting, which triggers a high-flux magmatic event (HFE) characterized by enriched isotopic signatures.

Crucially, this underthrusting mechanism drives material back toward the hinterland, resulting in magmatism that is not confined to the arc front but distributed across a widening arc. This relationship between arc migration and arc widening is illustrated in Figure 2 (igneous bedrock location vs. age plot). We recognize that the original multi-phase model in Figure 4 may have contributed to confusion by attempting to convey multiple processes simultaneously. In response, we have revised the figure to clarify that isotopic shifts are not restricted to the eastern arc front but also involve western hinterland magmatic systems within trailing, passively deforming segments of the thrust belt. This revision highlights the distributed nature of magmatic source evolution during orogenic cycles.

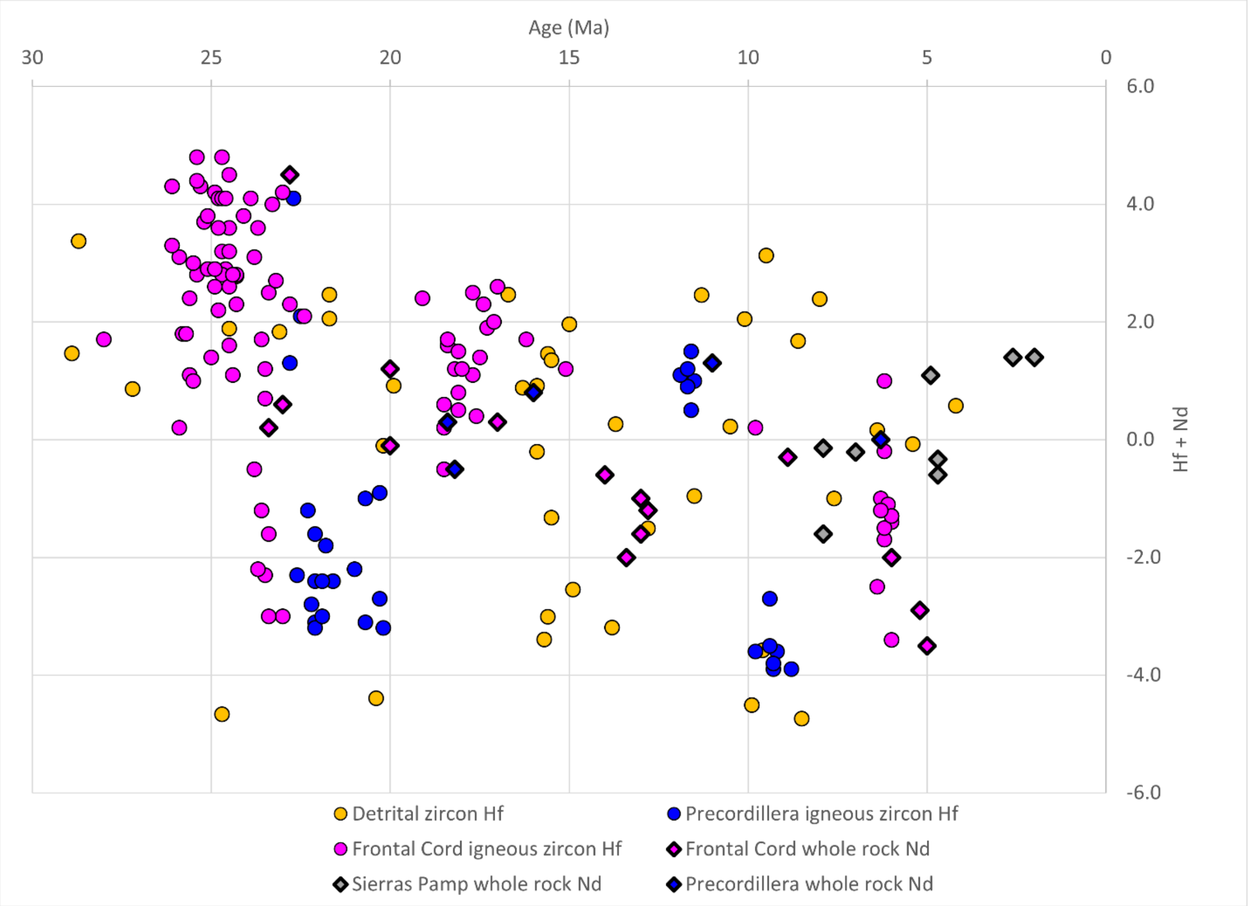

Figure A: Compiled zircon εHf (circle symbols) and whole-rock εNd (diamond symbols) data from the last 30 Myr in the southern Central Andes (see main text for references). Both Frontal Cordillera bedrock sample (pink symbols) and Precordillera (blue symbols) exhibit isotopic pull downs and pull ups that are superimposed on a general enrichment trend. Detrital zircon εHf (yellow circles) match the bedrock isotopic trends.

References Cited

Chapman, J.B., Ducea, M.N., Kapp, P., Gehrels, G.E. and DeCelles, P.G., 2017. Spatial and temporal radiogenic isotopic trends of magmatism in Cordilleran orogens. *Gondwana Research*, *48*, pp.189-204.

Kay, S.M. and Gordillo, C.E., 1994. Pocho volcanic rocks and the melting of depleted continental lithosphere above a shallowly dipping subduction zone in the central Andes. Contributions to Mineralogy and Petrology, 117(1), pp.25-44.

Kay, S.M. and Abbruzzi, J.M., 1996. Magmatic evidence for Neogene lithospheric evolution of the central Andean “flat-slab” between 30 S and 32 S. Tectonophysics, 259(1-3), p.15-28.

Kay, S.M., Mpodozis, C., Ramos, V.A. and Munizaga, F., 1991. Magma source variations for mid-late Tertiary magmatic rocks associated with a shallowing subduction zone and a thickening crust in the central Andes (28 to 33 S). Geological Society of America Special Paper, 265, p.113-137.

Kay, S.M., Mpodozis, C., Coira, B., 1999. Neogene magmatism, tectonism, and mineral deposits of the Central Andes (22° to 33°S latitude). In: Skinner, B.J. (Ed.), Geology and Ore Deposits of the Central Andes. Special Publication, No. 7. Society of Economic Geologists, p. 27–59.

Patchett, P.J., Kouvo, O., Hedge, C.E. and Tatsumoto, M., 1982. Evolution of continental crust and mantle heterogeneity: evidence from Hf isotopes. Contributions to Mineralogy and Petrology, 78(3), p. 279-297.

Vervoort, J.D., Patchett, P.J., Albarède, F., Blichert-Toft, J., Rudnick, R., Downes, H., 2000. Hf–Nd isotopic evolution of the lower crust. Earth and Planetary Science Letters, 181, p. 115–129.

Response to Reviewer #3

Provided below are the original reviewer comments followed by our detailed, point-by-point responses concerning the resubmitted manuscript to Nature Communications (NCOMMS-24-86680).
Our responses below are shown in **BOLD** and line numbers refer to the revised manuscript.

Reviewer 3 Comments

I read this paper and commented prior to reading the rebuttal to a previous review. In my first reading, I was very curious about the location of the data used in Figures 2 and 3 since they are not on Figure 1. This is important for readers to critically read the paper; the latitude and longitude of samples is reported in the supplementary material and figure 2 does include spatial information with the use of symbols. However, I recommend adding the sample locations used in Figure 3B to Figure 1. Below I expand on why this is necessary, especially in light of the previous reviews.

The zircon Hf and whole rock Nd trends are defined by the bedrock data. The detrital zircon Hf data broadly agree with the range of source regions, but take away from the well-defined bedrock pull-ups and pull-downs. In my opinion, the inclusion of the detrital data is fine for the overall interpretations, but is not as robust as the bedrock data. I comment below on the terminology around the detrital dataset at specific lines to help the authors rephrase some of their language around this.

The location of this data is central to the interpretations and differences between a reviewer and the authors. Given the implications of the proposed model to other convergent margins, linking Figure 1 to Figure 3 is essential for interpreting spatial trends. I had assumed the age of the circles and diamonds from Figure 3 (x-axis) correspond with the location of the data (Main Cordillera, Frontal, Pre, Sierras Pampeanas) as shown by the grey bars. However, the figure provided by Reviewer 2 clearly shows that some of the young (e.g., <10 Ma) zircons are from the Frontal Cordillera, not the regions farther east. For example, the Frontal Cordillera bedrock zircon Hf data at ~7 Ma range from ~ +1 to -3.5, and define the return to less evolved values (a "pull up" towards zero) on Figure 3B, but those data are outlined by the grey bar labeled Precordillera to the transition to the Sierras Pampeanas. At the same time, the whole rock Nd data from the Sierras Pampeanas support the pull up between ~8-3 Ma. This means that at <8 Ma within both the Frontal Cordillera and Sierras Pampeanas magmatism was moving towards 0, away from the evolved values recorded at ~ 10-8 Ma in the Precordillera. The mix of frontal cordillera and Sierras Pampeanas data with similar geochemical signatures alone does not support the conclusion that there is a progressively the spatial eastward trend, but does 1) support the model of progressively more evolved magmas through time and 2) when combined with the thermochronologic

data, can be used to support spatial trends.

In summary, the authors present a provocative model for arc magmatism along convergent margins that will promote future interrogation of these margins and the model itself. This is a contribution that merits publication. However, I highly recommend the authors make use of variable symbols/colors for their bedrock and detrital samples so that the readers know the spatial distribution. This is done for the AFT and AHe data on Figure 2 well, but these locations are not shown on Figure 1. For the AFT and AHe data, a supplementary location map would suffice. However, given the emphasis on the Hf and Nd data in Figure 3, adding these data to Figure 1 and symbolizing the four geographic regions with different colors would result in a more transparent, and ultimately, more useful visual of the data to support the authors interpretations.

The authors are free to email me if any of my suggestions are unclear.

Best Wishes,
Devon

Response to Reviewer 3

We appreciate Reviewer 3's thoughtful assessment of the manuscript in its current form and their consideration of how it has evolved through previous review rounds. We agreed with their suggestion to include sample locations for the whole-rock εNd and zircon εHf on the Figure 1B geologic map, using distinct symbology to differentiate tectonic provinces and sample types. We have updated both Figure 1 accordingly and applied the same symbology to the age vs geochemistry plot in Figure 3B to better illustrate the spatial distribution of Andean arc geochemical signatures. Both of the figures have been revised to incorporate these additions. See new Figures 1 and 3.

Specific Comments:

Line 44-45: Consider re-phrasing to be more direct. E.g., We hypothesize that short-term orogenic phases are linked to shifts in thermal…

We have modified the sentence to highlight our hypothesis.

Line 115: "consistent" is strong here considering the many outliers from the detrital record (Figure 3B). I recommend simply removing "consistent". There is a broad geochemical match.

If one removes the DZ EHf record, the bedrock record nicely shows the pull downs and pull-ups, albeit a bit more tightly constrained. Therefore, the emphasis should be placed

on the bedrock record, with the detrital supporting interpretations, not broadening.
We agree, and have removed "consistent" from the statement.

Line 166: Thermochronometric dates.
Good catch, we changed "thermochronometry" to "thermochronometric"

Lines 177-179 speak to shortening in the latest Oligocene-early Miocene, yet the curves in Panel C do not start until 20 Ma and there is no information on orthogonal subduction in panels B or C. Therefore, reference to this figure here could be clarified or removed. More importantly, panel B nicely shows the rapid increase in convergence rate at 24-23 Ma, which corresponds to the inboard shift in magmatism. This is crucial and not discussed in the text.
We have rephrased the statement to better reflect the data plotted in Figure 2B.

AHe reported data: The main text and figure caption must state these are mean dates from detrital and igneous bedrock datasets. This is crucial because it is a simplification of the data. Most samples have enormous ranges of single grains (e.g., AN01 has between 41-68 Ma, but a mean of 61…continuing down Supplementary File S4, many thermochronologists would argue these data should only be reported as single grain ages. I suspect if these are plotted, some of the trends dissipate. However, I understand the authors aim to share a ton of data clearly on Figure 2. Therefore, I recommend adding a supplementary figure to S4 that shows all the single age grains to eliminate any criticism that use of mean AHe is misleading. For quite a few AHe samples, the mean age is meaningless given the range of dates and control on those ages from geologic and mineral specific sources of error.
We have added "mean AHe" and "Central AFT" to Figure 2A legend and expanded the methods section to clarify the data types included in our regional compilation. Additionally, we have created Supplemental Figure 3 that compares single grain age with mean age AHe and central age AFT. The eastward younging in thermochronology age holds up in both single age and mean age sample plots (see new Supplemental Figure 3).

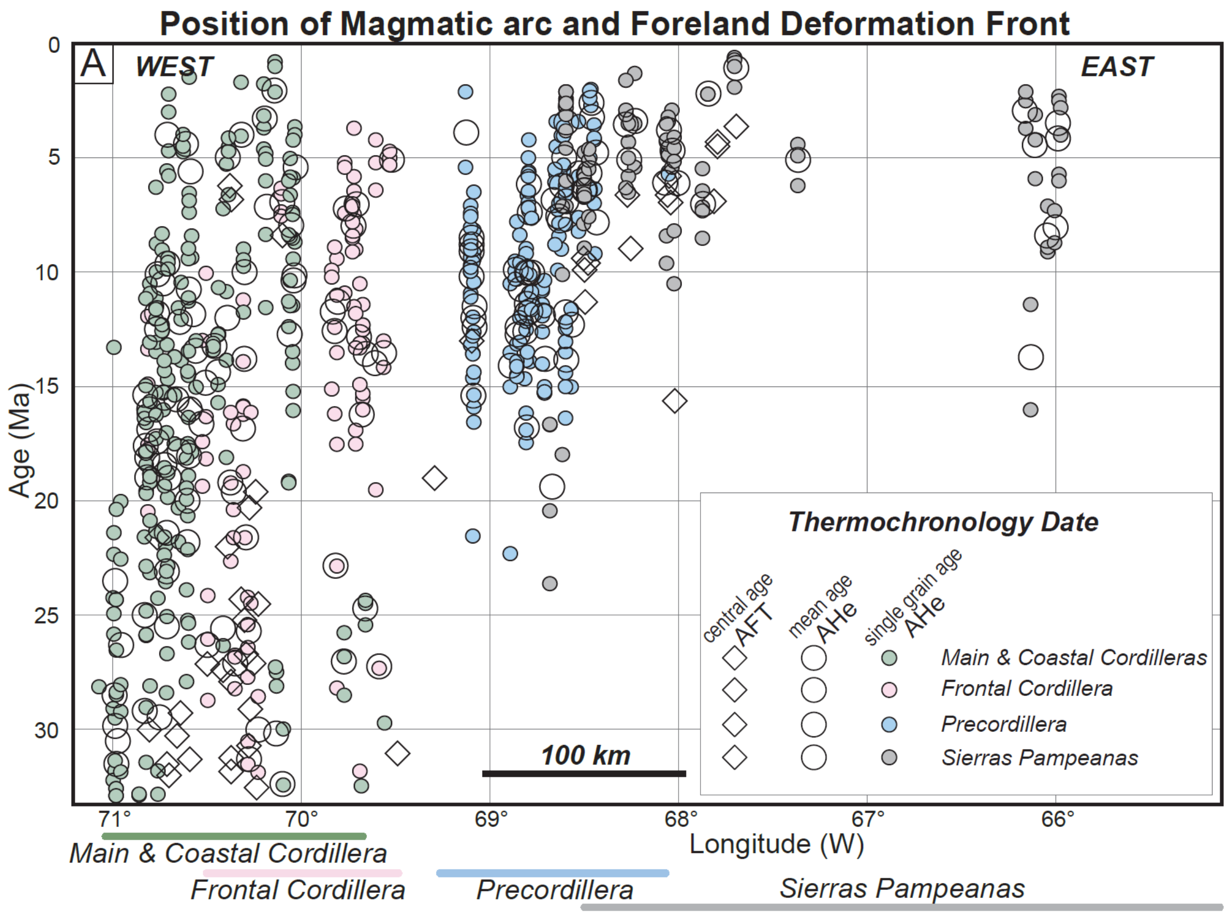

Supplemental Figure 3: Time-distance plot of thermochronological dates at 27-33 S for single grain age apatite (U-Th)/He dates (color circles) compared to mean age AHe and central apatite fission track dates.

Figure Comments

Figure 2: The “arc front” polygon is a bit confusing when reading along with the text, since the data extent eastward of this. For example at 25-23 Ma, there is magmatism east of the “arc front”. Therefore, what does this line/polygon represent? It seems to be an oversimplication that is not needed. If used, it should mimic the text description of this plot over Lines 122-136.
You make a great point. We have modified Figure 2 arc front polygon to better reflect the changes in position outlined in the main text. See new Figure 2.

Figure 2: At first, I thought the data came from the labeled basins, but the data extend into the main and coastal Cordillera. Once I got to line 184, it became the basin subsidence records of these areas are why they are labeled on Figure 1. Therefore, I am left wondering where the data are spatially in the study area? I highly recommend

adding symbols on figure 1 that show where all the data from figure 2 are spatially located. Alternatively, this could be a supplemental figure.

We have created two supplemental figures that plot the location of igneous geochronology samples (Supplemental Figure S1) and location of detrital and bedrock thermochronology samples overlain a DEM hill shade basemap. We have added text in the Methods section explaining these plots.

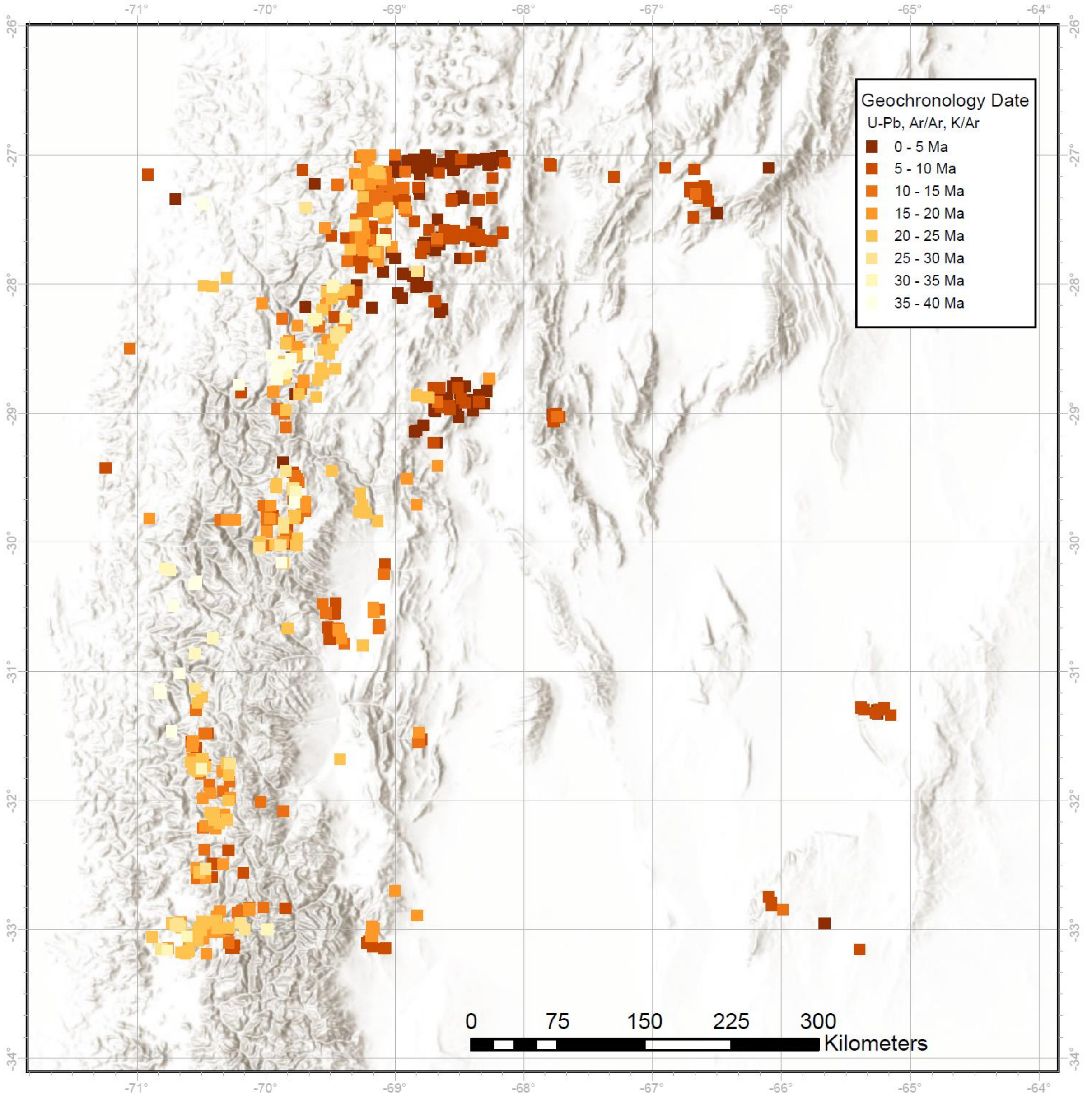

Supplemental Figure 1: Spatial and temporal variation in Andean arc magmatism. Data compilation of U-Pb, Ar/Ar and K/Ar ages from Pilger, 2024.

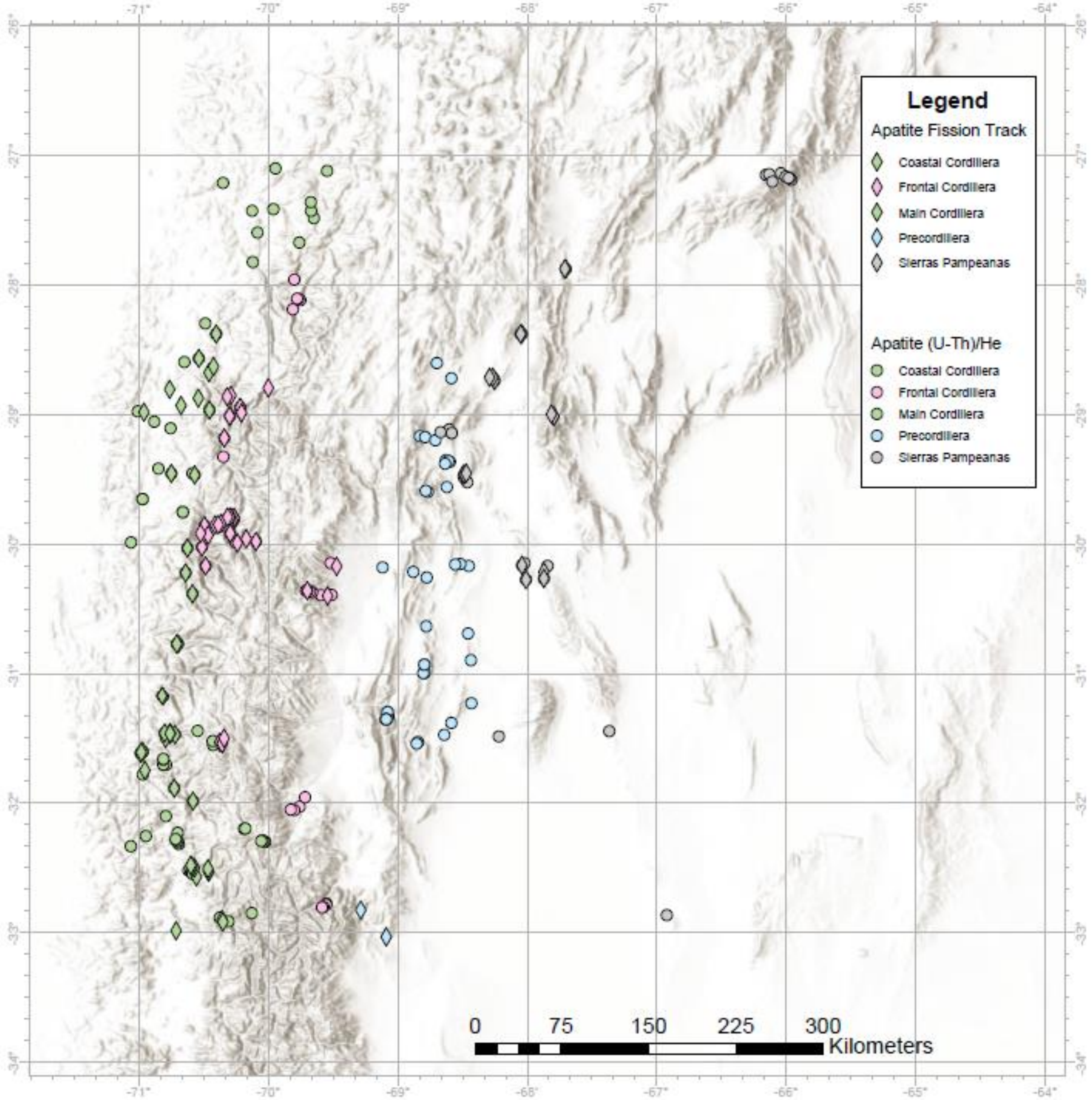

Supplemental Figure 2: Spatial distribution in Andean thermochronological dates. Data compilation of apatite (U-Th)/He dates from: Lobens et al., 2013; Bense et al., 2013; Levina et al., 2014; Hoke et al., 2015; Fosdick et al., 2015; Ortiz et al., 2015, 2021; Lossada et al., 2017; Piquer et al., 2017; Rodriquez et al., 2018; Stevens-Goddard and Carrapa, 2018; Maydagan et al., 2020; Mackaman-Lofland et al., 2020, 2022, 2024; Stalder et al., 2020; Plonka et al., 2023. Data compilation of apatite fission track dates from: Coughlin et al., 1998; Avila et al., 2005; Farias et al., 2008; Sato et al., 2015; Fosdick et al., 2015; Murillo et al., 2017; Rodriguez et al., 2018; Stevens-Goddard and Carrapa, 2018; Maydagan et al., 2020; Stalder et al., 2020; Ortiz et al., 2021.

Figure 2: The caption needs to state these are single mean AHe ages and central (?) AFT ages. Note: I assume these are central AFT and not pooled ages, but both the

main text and supplementary table S5 need to make this clear.
We have added “mean AHe” and “Central AFT” to Figure 2A legend. See new Figure 2.

Figure 3: Caption for c, please add “detrital” before zircon U-Pb ages. The label on panel C is quite small and this was unclear
Added “detrital” to the figure caption. See new Figure 3.

Figure 4: This is well-crafted figure! However, it was unclear from the figure alone or caption that this is a proposed sequence until reading the discussion. To make this figure stand-alone (i.e. the citable figure that others may use in presentations, etc.), I recommend adding some sort of text (e.g., t1, t2, t3, and t4) or an arrow linking them (younger ----- > older).
We have added ”Step” labels to each panel of the figure to highlight hypothesized time scale for each step in the multi-phase model. See new Figure 4.

Dear Editor,

In this letter I send you my comments on the revised version of the paper entitled "Inboard advance of arc magmatism regulates mountain building in the Andes" coauthored by Capaldi, T., Horton, B., Mackaman-Lofland, C., Fuentes, F. and Ortiz, G.

In this new version of the manuscript, the authors have taken care of all the minor comments I made in my previous review, yet our disagreement with the interpretation of the Hf isotopic data remains. I see two main issues in this discussion: (i) the scopes and limitations of data from detrital zircons, and (ii) the interpretation of the ƐHf(t) data in terms of the spatial-temporal-compositional analysis. I refer to each of these in the following.

Regarding the detrital zircon data, in my previous review I questioned its use in the model presented by the authors as they have no control over the precise original location of such data in space in the Andean orogen. The authors have replied that it has been shown that this is still a robust tool in tracking the timing and pattern of magmatism. They also add that the data fill in the gaps left by the "petrologist effect" (in bedrock sampling non-altered igneous units) and that it efficiently samples large areas of catchment bedrock sources. I agree with all these statements, yet still the core of my initial critic remains: the detrital data cannot be used for spatial interpretations about the arc, regarding its location and composition at any given time, because there is no control on the original location of such data. I include below a graph of ƐHf(t) vs t diagram (Fig. A) using the data presented by the authors where the detritic data has been separated from the bedrock data in the symbology (small gray dots). As indicated by the authors, the detrital data indeed totally overlaps with the bedrock data and I agree that those zrcs probably come from the Precordillera and the Frontal Cordillera areas. The issue here is that there is no precise control of where those zrcs come from among and within the mentioned areas. The model presented by the authors is fully dependent on a spatial variable, in the location and composition of the arc magmas in time, so detrital data cannot be used for supporting inferences in that respect.

Regarding the interpretations of the ƐHf(t) data, the model presented by the authors predicts a cycle that: (1) starts with arc magmas at a given location with high ƐHf (mantle-like), (2) then the arc magmas show a decreasing ƐHf (more crustal-like) at that same location, (3) then the arc migrates eastwards and magmas show a high ƐHf (mantle-like) starting the cycle again. This model is clearly depicted in Fig. 4 of the manuscript in the previous and the current version of this work. In my previous review I argued against this interpretation based on the actual location of the samples recording the arc location and composition in time (bedrock samples) along with considering previous works in the area. I indicated that Cenozoic igneous rocks in this region show more enriched signatures (more "crustal like") towards the east, as shown by mostly coeval units emplaced in the Frontal Cordillera and the Precordillera. This was ascribed by Jones et al. (2015), and previously by Kay & Abbruzzi (1996), to the presence of an old basement in the eastern regions (Grenvillian Basement – Cuyania Terrane?) responsible to imprint the enriched signature to the magmas that have transversed it in the eastern region (Precordillera). In their reply, the authors recognize that in this region Andean magmas show progressively more enriched compositions towards the east, but they question my arguments that the ƐHf(t) trend is purely recording a spatial trend indicating that this mechanism alone

cannot fully explain the temporal variations observed in the data. Regarding this, there are some misinterpretations on my comments: I do argument that there is a spatial control on the composition of arc magmas, they become generally more enriched towards the east, but this is in addition over imprinted by the general increase in crustal thickness throughout the Andes construction in Cenozoic (from ~22-18 Ma to the present; see Giambiagi & Ramos, 2002; Farías et al., 2010) which adds an increasing crustal signature to progressively younger magmas (lower ƐHf(t) signatures). The ƐHf(t) vs t diagram of Fig. A below shows this very clear for the Frontal Cordillera: igneous rocks emplaced there define a trend towards enriched signatures in time. Moreover, I have included below a map showing the location of all samples discussed in the Capaldi et al. study (detrital and bedrock; Fig. B), where it can be seen that the "trend" for the Frontal Cordillera is constrained by samples very restricted in space (westernmost samples of all those considered in this study; blue dots in Fig. A, B). Regarding rocks emplaced towards the east (in the Precordillera and Precordillera/Frontal Cordillera), as I mentioned in my previous review, they also define a trend towards more enriched ƐHf(t) signatures in time but at a comparatively lower ƐHf values (more enriched than those of the Frontal Cordillera) with minor juvenile inputs.

I must refer to two arguments the authors presented in their reply to discredit the interpretations I discussed above (Jones et al., 2015; Kay & Abbruzzi, 1996). First, they argue that the Jones et al. (2015) dataset is spatially limited to -70°S to -69°S: samples were collected mainly in the Frontal Cordillera with one sample (RJ11A5) being truly in the Precordillera across the speculated Chilenia-Cuyania terrane boundary. I'm not sure where the authors point with this argument: the Jones et al. (2015) data is the only data with spatial location control they have for their model, discrediting this dataset discredits their own spatial-temporal-compositional interpretations. Moreover, the detrital data they present does not add much to the story, given its location and the arguments I presented before regarding such data (see Fig. A, B). Whether limited or not, the Jones et al. (2015) data shows clearly the trends I discussed in the previous paragraph and that are illustrated in Fig. A below. The second argument presented by the authors is that the variability of average zircon ƐHf in the Frontal Cordillera over the past 300 Myr ranges between +4 and -4 in ƐHf space (Capaldi et al., 2021- Fig. 10). I agree, the Fig A below shows this clearly for the Cenozoic: magmas that have transversed the Frontal Cordillera area show a full range of ƐHf(t) values between -4 and +4. The issue here is that, within this range, during Cenozoic there is a temporal evolution to lower signatures (more crustal-like) in younger ages, which is most likely related to the increasing crustal thickness that accompanies the construction of the modern orogen.

In their reply, the authors also present their interpretation of the Jones et al. (2015) data in detail, indicating that samples in the Frontal Cordillera exhibit isotopic pull-downs (samples PC14, RJ11A10, RJ11A11, RJ11A14 between 24-21 Ma; samples RJ11A17, RJ11A15, DI095, MQ33 between 10-6 Ma) and pull-ups (samples SP80, 1026, RF62 between 19-17 Ma; and sample RJ11A7 at 12 Ma). The problem here is what they are considering as located in the Frontal Cordillera or elsewhere. Setting aside "names", Figs. A and B below show that within the full age range considered the spatially restricted group of westernmost samples (in blue in the figures) is comparatively more depleted (mantle-like) than coeval magmas emplaced in eastern regions except for local juvenile inputs (2 samples at ~24 and 12 Ma).

The authors have added to the new version of the manuscript a paragraph that describes general aspects of the arc magmas composition in the studied region. They indicate there that: “Coupled zircon geochemical and age data exhibit a progressive decrease in zircon εHf values over the last 30 Myr (Fig. 3B). When considered alongside with bedrock sample locations (Fig. 2A), a spatial relationship emerges: relatively asthenospheric (juvenile) compositions are preferentially located near the trench, in contrast while more lithospheric (evolved) contributions are concentrated toward the craton (Fig. 3B). Superimposed on this 30 Myr trend are higher-frequency phases (5-8 Myr) in which the relatively depleted positive εHf values represent background arc magmatism generated by juvenile mantle sources, which were perturbed during isotopic pull-downs phases reflecting greater contribution of evolved crustal sources (Fig. 3B). A 1 to 2 Myr temporal overlap in both depleted and enriched values are observed at the onset of the isotopic pull-down indicating magma mixing processes during changes in magmatic source (Hawkesworth and Kemp, 2006)”. The processes described here, and the model depicted in Fig. 4 of the manuscript, are not supported by the data. For example, there is not a single sample from the spatially restricted westernmost group of the Fontal Cordillera that describes an “isotopic pull down” from the general trend they described through Cenozoic (blue spots in Fig A); the comparatively lower ƐHf signatures are described exclusively by samples located to the east. Moreover, the model presented by the authors predicts a zig-zag ƐHf trend marked by high and low values developing sequentially in time towards the east: this is by no means described by data with spatial control (Fig A, B).

In summary, I consider that the model presented by the authors is attractive and can explain several geological observations. However, in my opinion the magmatic evolution this model entails, in terms of the sequential variation in arc location and its composition, is not supported by the data.

Best regards.

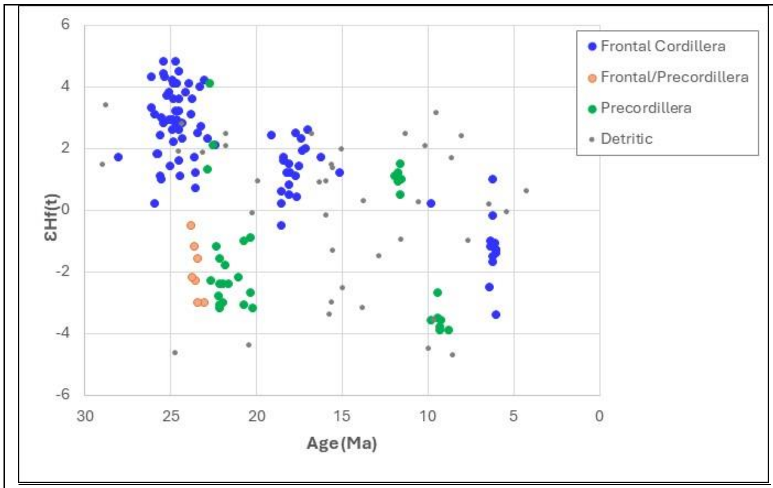

Fig A. ƐHf(t) vs Age diagram for zircon data presented in the manuscript. Data has been separated in: (i) sizes according to whether they belong to bedrocks or detrital zircons (large vs small dots), and (ii) their location in the Andean orogen for bedrock data (according to the classification of Jones et al., 2015, where this data originally come from). Original data is from Jones et al. (2015) and Capaldi et al. (2021).

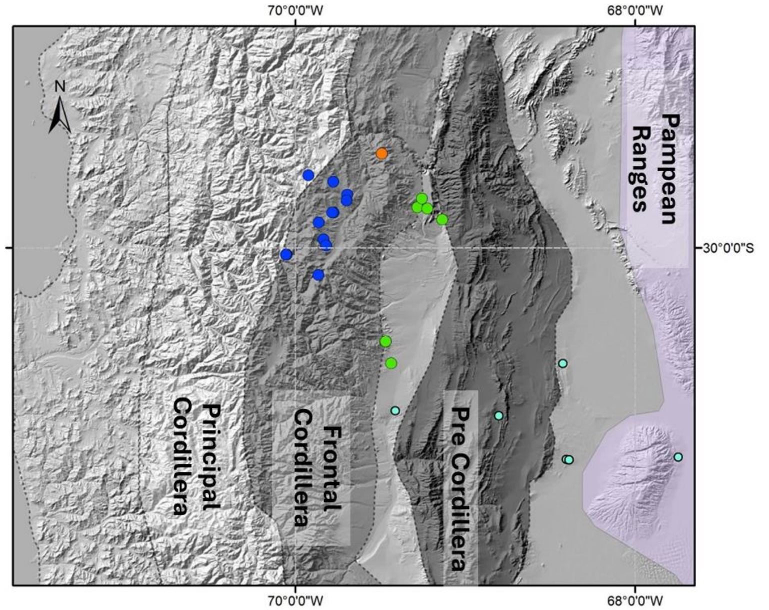

Fig B. Spatial location of the samples displayed in the ƐHf(t) vs Age diagram of Fig. A. Shaded areas in different tones highlight the main morphostructures of the Andean orogen in this area. The detrital data is indicated in the small cyan dots, while the bedrock data is indicated in the same blue, orange and green dots displayed in the ƐHf(t) vs Age diagram of Fig. A. These colors correspond respectively to the Frontal Cordillera, Frontal/Precordillera, and Precordillera, as originally assigned by Jones et al. (2015).

References

Capaldi, T.N., McKenzie, N.R., Horton, B.K., Mackaman-Lofland, C., Colleps, C.L. and Stockli, D.F., 2021. Detrital zircon record of Phanerozoic magmatism in the southern Central Andes. Geosphere, 17(3), p.876-897.

Farías, M., Comte, D., Charrier, R., Martinod, J., Tassara, A., Fock, A., 2010. Crustal-scale structural architecture of the central Chile Andes based on 3D seismic tomography, seismicity, and surface geology: implications for mountain building in subduction zones. Tectonics 29, TC3006.

Giambiagi, L.B., Ramos, V.A., 2002. Structural evolution of the Andes in a transitional zone between flat and normal subduction (33°30'-33°45'S), Argentina and Chile. Journal of South American Earth Sciences 15, 101-116.

Jones, R.E., Kirstein, L.A., Kasemann, S.A., Dhuime, B., Elliott, T., Litvak, V.D., Alonso, R., Hinton, R. and Facility, E.I.M., 2015. Geodynamic controls on the contamination of Cenozoic arc magmas in the southern Central Andes: Insights from the O and Hf isotopic composition of zircon. Geochimica et Cosmochimica Acta, 164, p.386-402.

Kay, S.M. and Abbruzzi, J.M. Magmatic evidence for Neogene lithospheric evolution of the central Andean "flat-slab" between 30°S and 32°S. Tectonophysics, 259, 15-28.
